# Monocyte-derived dendritic cells link localized secretory IgA deficiency to adaptive immune activation in COPD

**DOI:** 10.1038/s41385-020-00344-9

**Published:** 2020-09-23

**Authors:** Bradley W. Richmond, Samira Mansouri, Ana Serezani, Sergey Novitskiy, Jessica B. Blackburn, Rui-Hong Du, Hubaida Fuseini, Sergey Gutor, Wei Han, Jacob Schaff, Georgii Vasiukov, Matthew K. Xin, Dawn C. Newcomb, Lei Jin, Timothy S. Blackwell, Vasiliy V. Polosukhin

**Affiliations:** 1 Department of Veterans Affairs Medical Center, Nashville, TN USA; 2 Division of Allergy, Pulmonary, and Critical Care Medicine, Vanderbilt University School of Medicine, Nashville, TN USA; 3 Department of Medicine, Division of Pulmonary, Critical Care, and Sleep Medicine, University of Florida College of Medicine, Gainesville, FL USA

## Abstract

Although activation of adaptive immunity is a common pathological feature of chronic obstructive pulmonary disease (COPD), particularly during later stages of the disease, the underlying mechanisms are poorly understood. In small airways of COPD patients, we found that localized disruption of the secretory immunoglobulin A (SIgA)-containing mucosal immunobarrier correlated with lymphocyte accumulation in airway walls and development of tertiary lymphoid structures (TLS) around small airways. In SIgA-deficient mice, we observed bacterial invasion into the airway epithelial barrier with lymphocytic infiltration and TLS formation, which correlated with the progression of COPD-like pathology with advanced age. Depletion of either CD4^+^ or CD8^+^ T lymphocytes reduced the severity of emphysema in SIgA-deficient mice, indicating that adaptive immune activation contributes to progressive lung destruction. Further studies revealed that lymphocyte infiltration into the lungs of SIgA-deficient mice was dependent on monocyte-derived dendritic cells (moDCs), which were recruited through a CCR2-dependent mechanism in response to airway bacteria. Consistent with these results, we found that moDCs were increased in lungs of COPD patients, along with CD4^+^ and CD8^+^ effector memory T cells. Together, these data indicate that endogenous bacteria in SIgA-deficient airways orchestrate a persistent and pathologic T lymphocyte response through monocyte recruitment and moDC differentiation.

## Introduction

The airways are continuously exposed to endogenous and inhaled microbes and noninfectious irritants. To prevent these stimuli from damaging the lung directly or indirectly through activation of inflammatory responses, the respiratory epithelium continuously maintains a frontline defense barrier on the airway surface. Inhaled microorganisms and environmental microparticles are trapped by surface mucus, inactivated or destroyed by soluble enzymatic and antimicrobial factors, agglutinated by antigen-specific mucosal immunoglobulins, and ultimately cleared from the airway via the mucociliary escalator.^1,2^ When environmental agents traverse the frontline defense barrier and stimulate host cells, innate and/or adaptive immune responses, which represent second and third lines of defense, are activated to ensure elimination of invaders. However, unlike the surface immunobarrier, inducible innate and adaptive immune responses are often accompanied by tissue damage that must be repaired for homeostasis to return.

Secretory immunoglobulin A (SIgA) is the dominant mucosal immunoglobulin at the airway surface and a core component of this frontline defense system.^3,4^ Production of SIgA begins in the lamina propria where subepithelial plasma cells produce two IgA monomers joined by a short polypeptide called the joining (J) chain.^5^ Dimeric IgA (dIgA) binds covalently via the J chain to the polymeric immunoglobulin receptor (pIgR) on the basolateral surface of airway epithelial cells.^6^ After binding, pIgR/dIgA complexes are endocytosed and transported within endosomes to the apical surface.^7^ There, pIgR is proteolytically cleaved releasing dIgA and the extracellular portion of pIgR (the secretory component or SC) into the airway lumen to form SIgA.

Chronic obstructive pulmonary disease (COPD) is a common, debilitating, and often fatal lung disease associated with inhalation of noxious substances, particularly tobacco smoke.^8^ In COPD patients, structural abnormalities in the airway epithelium are associated with functional defects, including loss of the SIgA immunobarrier.^9–12^ Consistent with its established role in mucosal homeostasis, loss of the SIgA immunobarrier in COPD airways is associated with chronic bacterial invasion, neutrophilic inflammation, and more severe airway pathology.^9,10,12^ Loss of SIgA in the airways of COPD patients results from decreased pIgR expression in the airway epithelium, which prevents SIgA transcytosis despite increased numbers of IgA-producing plasma cells.^10–13^ Mice lacking pIgR (*pIgR*^*−/−*^ mice) are SIgA deficient and develop persistent inflammation in the lungs, along with progressive emphysema and small airway remodeling that resemble the pathology of patients with COPD.^14,15^ Although existing data suggest that loss of the SIgA immunobarrier plays a causative role in COPD, it remains unknown whether SIgA deficiency contributes to adaptive immune activation, which is common in lungs of patients with advanced COPD.^16–24^

We investigated connections between loss of the SIgA immunobarrier and persistent activation of adaptive immunity in COPD patients and *pIgR*^*−/−*^ mice. We found that disruption of the SIgA immunobarrier initiates a cycle of pathologic cross-talk between the innate and adaptive branches of the immune system that is coordinated by monocyte-derived dendritic cells (moDCs). These studies suggest that loss of the frontline SIgA defense barrier is a fundamental defect driving adaptive immune activation in COPD.

## Results

### Loss of the SIgA immunobarrier causes adaptive immune activation

**Fig. 1:**
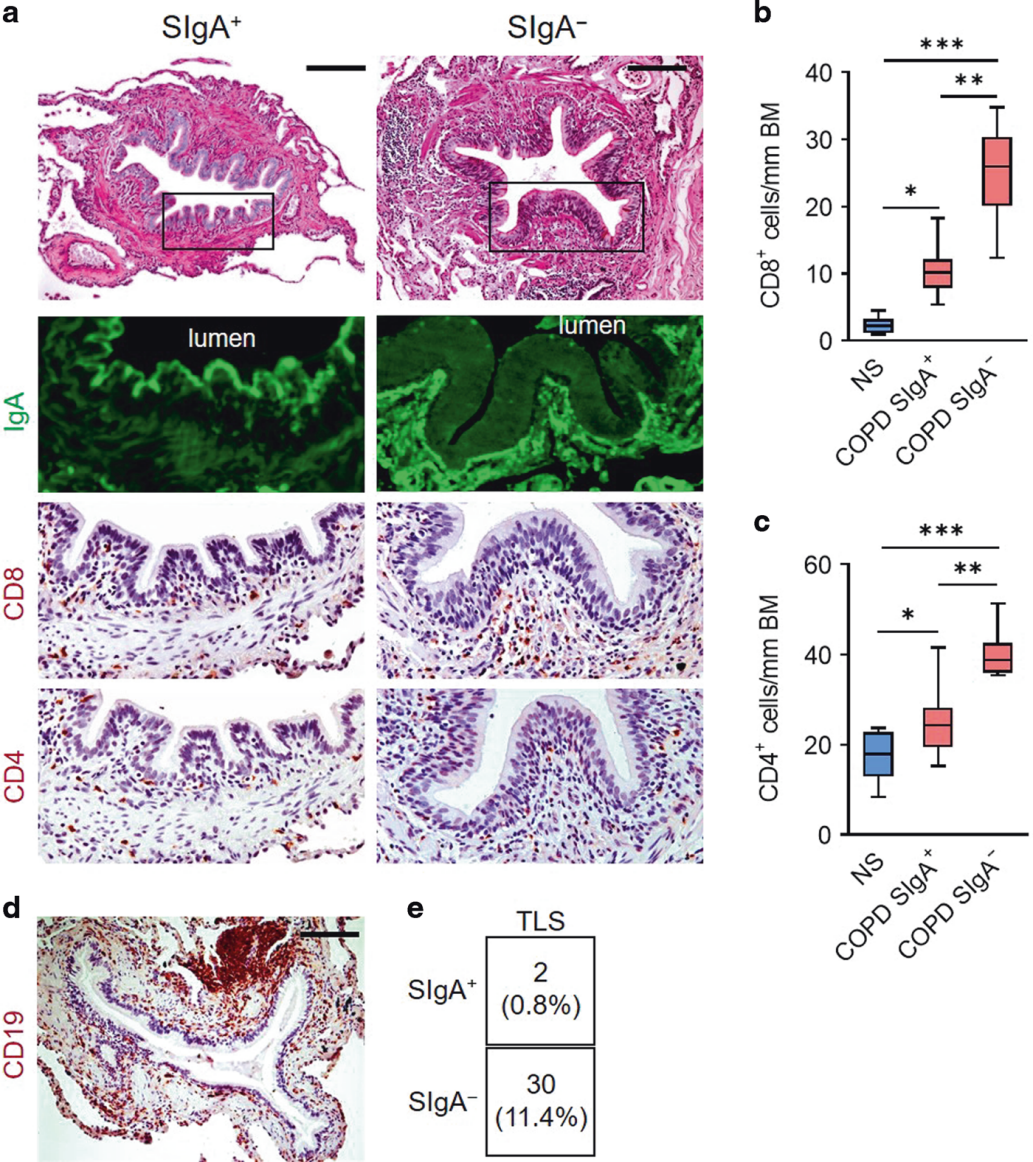
T and B lymphocytes preferentially accumulate around SIgA-deficient airways in patients with COPD. **a** Immunostaining for SIgA (green) and CD4 or CD8 (brown) in 5 μm lung sections from a COPD patient. Scale bar = 100 μm. **b**, **c** Quantification of CD8^+^ (**b**) or CD4^+^ (**c**) T lymphocytes/mm basement membrane in 5 μm lung sections from COPD patients according to airway SIgA status and lifelong non-smokers (NS). Small airways from COPD patients were classified as SIgA^+^ or SIgA^-^ based on the quantification of SIgA immunofluorescence. **d** Example of a tertiary lymphoid structure in a COPD patient as determined by immunostaining for CD19. Scale bar = 100 μm. **e** Table illustrating the number and percentage of TLS according to airway SIgA status. **b** *p < 0.01 compared to NS, **p < 0.0001 compared to COPD SIgA^+^, ***p < 0.0001 compared to NS, (ANOVA). **c** *p < 0.05 compared to NS, **p < 0.001 compared to COPD SIgA^+^ airways, ***p < 0.0001 compared to NS (ANOVA). Box-and-whisker plots represent median, interquartile range, and range.

To examine the relationship between localized SIgA deficiency and lymphocyte accumulation in COPD airways, we obtained lung sections from 12 patients undergoing transplantation for advanced COPD and 8 lifelong nonsmokers (NS) whose lungs were rejected for lung transplantation (Supplementary Table 1). After classifying small (<2 mm diameter) airways from COPD patients as SIgA-deficient (SIgA^-^) or SIgA-replete (SIgA^+^) based on immunostaining for SIgA on the luminal surface as previously described,^12^ we determined the number of CD8^+^ and CD4^+^ T lymphocytes present in the airway wall (Fig. 1a). Although SIgA^+^ airways from COPD patients had more CD8^+^ and CD4^+^ T lymphocytes than lifelong nonsmokers, the highest numbers of cells were in SIgA^-^ airways from patients with COPD suggesting loss of SIgA is associated with recruitment of T lymphocytes to small airways.

In addition to infiltration of airway walls, lymphocytes can accumulate in TLS adjacent to small airways in patients with severe COPD.^13,20^ Therefore, we examined the relationship between TLS formation (identified by immunostaining for CD19, Fig. 1d) and the SIgA status of each adjacent airway. In 263 airways examined, 11.4% of SIgA^-^ airways had adjacent TLS whereas TLS were rarely observed around SIgA^+^ airways (0.8%) (Fig. 1e). Overall, 54.8% of small airways in this group of patients with severe COPD were SIgA^-^ but 93.1% of TLS were nearest a SIgA^-^ airway, indicating a marked over-representation of TLS adjacent to airways with mucosal immune dysfunction.

**Fig. 2:**
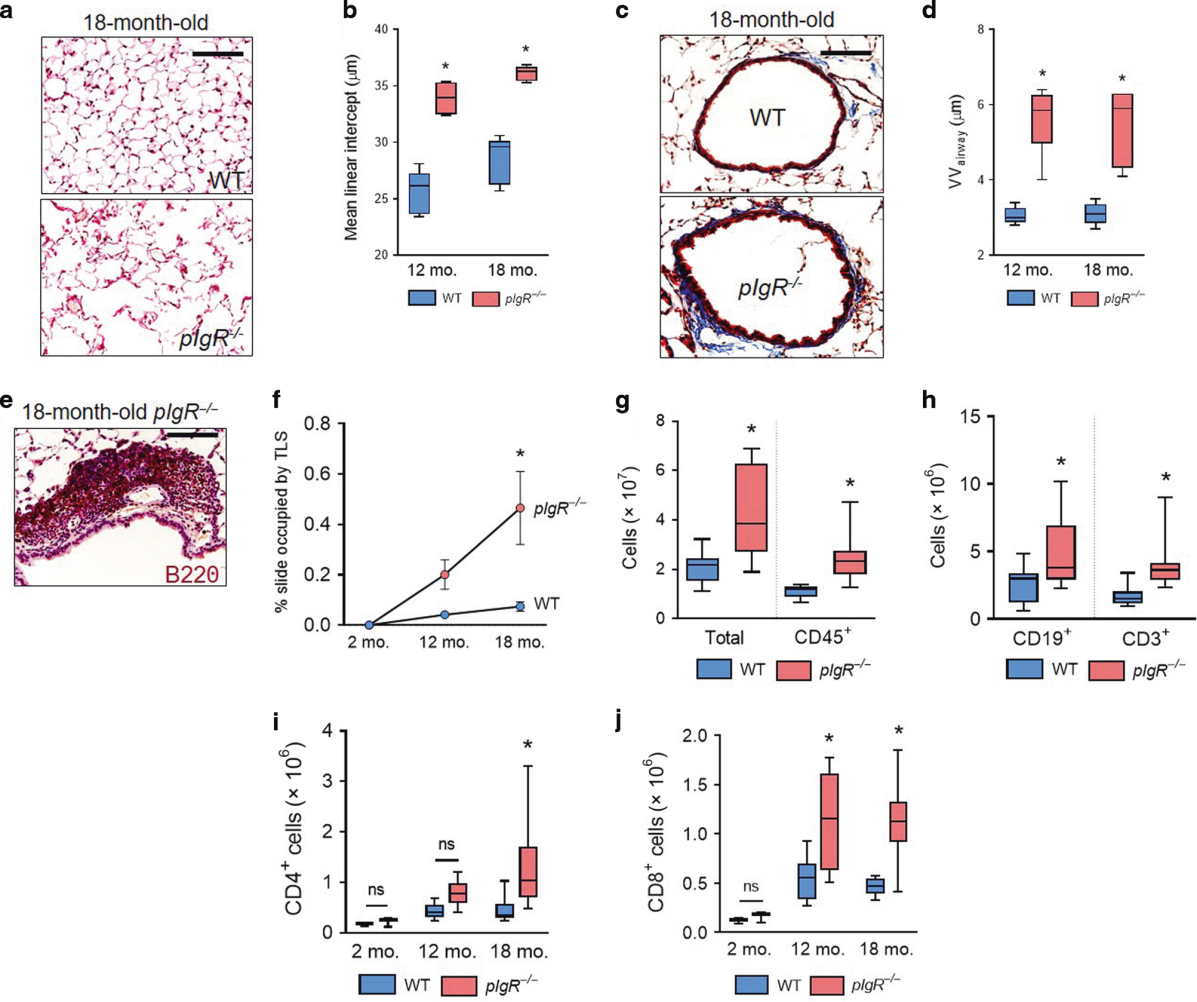
*pIgR*^−/−^ mice spontaneously develop COPD-like lung pathology and adaptive immune activation. **a** Representative image of emphysema in an 18-month-old *pIgR*^*−/−*^ and age-matched WT mouse (hematoxylin and eosin, scale bar = 50 μm). **b** Measurement of mean linear intercept (MLI), a morphometric measurement of emphysema, in WT and *pIgR*^−*/−*^ mice at the indicated ages. **c** Representative image of small airway wall thickening in an 18-month-old *pIgR*^*−/−*^ and age-matched WT mouse (Masson’s trichrome, scale bar = 50 μm). **d** Measurement of VV_airway_, a morphometric measurement of small airway wall thickness, in WT and *pIgR*^−*/*−^ mice at the indicated ages. **e** Example of a tertiary lymphoid structure (TLS) in an 18-month-old *pIgR*^−*/−*^ mouse as indicated by immunostaining for B220. Scale bar = 50 μm. **f** Morphometric analysis of TLS area in lungs of WT or *pIgR*^−*/−*^ mice at the indicated ages. **g**–**j** Quantification of total, CD45^+^, CD19^+^, CD3^+^, CD4^+^, and CD8^+^ cells in the lungs of 18-month-old WT and *pIgR*^−*/−*^ mice by flow cytometry. **b**, **d** *p < 0.0001 compared to age-matched WT mice (2-way ANOVA with Bonferroni post hoc test); *n* = 5–6 mice group. **f** *p < 0.001 com*p*ared to 18-month-old WT mice (2-way ANOVA with Bonferroni post hoc test); *n* = 3 mice/group. **g** *p < 0.05 compared to 18-month-old WT mice (*t*-test); *n* = 9–12 mice/group. **h** *p < 0.05 compared to 18-month-old WT mice (Mann–Whitney test); *n* = 9–12 mice/group. **i**, **j** *p < 0.01 compared to age-matched WT mice (2-way ANOVA with Bonferroni post hoc test); *n* = 3–12 mice/group. Box-and-whisker plots represent median, interquartile range, and range.

We next evaluated whether loss of the airway SIgA immunobarrier induces lymphocyte accumulation in SIgA-deficient (*pIgR*^*−/−*^) mice. Similar to patients with COPD, we observed increased emphysema and small airway wall thickening in *pIgR*^*−/*−^ mice as they aged (Fig. 2a–d). TLS were evident in aged (18-month-old) *pIgR*^−*/*−^ mice adjacent to small airways (Fig. 2e); however, TLS were absent in young adult (2-month-old) wild-type (WT) and *pIgR*^*−/−*^ mice and were rare in WT mice at any age (Fig. 2f).

In lungs of aged *pIgR*^−*/−*^ mice, total CD45^+^ immune/inflammatory cells measured by flow cytometry were increased compared to WT mice (Fig. 2g), consistent with previous data.^14,15^ Analysis of lymphocyte subsets showed that B (CD19^+^) cells and T (CD3^+^) cells were increased in aged *pIgR*^*−/−*^ mice, suggesting adaptive immune activation in the lungs (Fig. 2h). While lungs of *pIgR*^*−/−*^ and WT mice had similar numbers of T lymphocytes at 2 months of age, *pIgR*^*−/−*^ mice developed increased numbers of CD4^+^ and CD8^+^ T lymphocytes in the lungs as they aged (Fig. 2, i and j). Additionally, we observed a trend toward increased numbers of CD4^+^/IL-17^+^ cells in the lungs of aged *pIgR*^*−/−*^ mice, while no differences were observed in CD4^+^/IFN-γ^+^ cells, γδ T cells, or NK cells. (Supplementary Fig. 1). Together, these data indicate that loss of the SIgA immunobarrier is associated with adaptive immune activation in COPD.

### T lymphocytes contribute to COPD-like pathology in lungs of *pIgR*^*−/−*^ mice

**Fig. 3:**
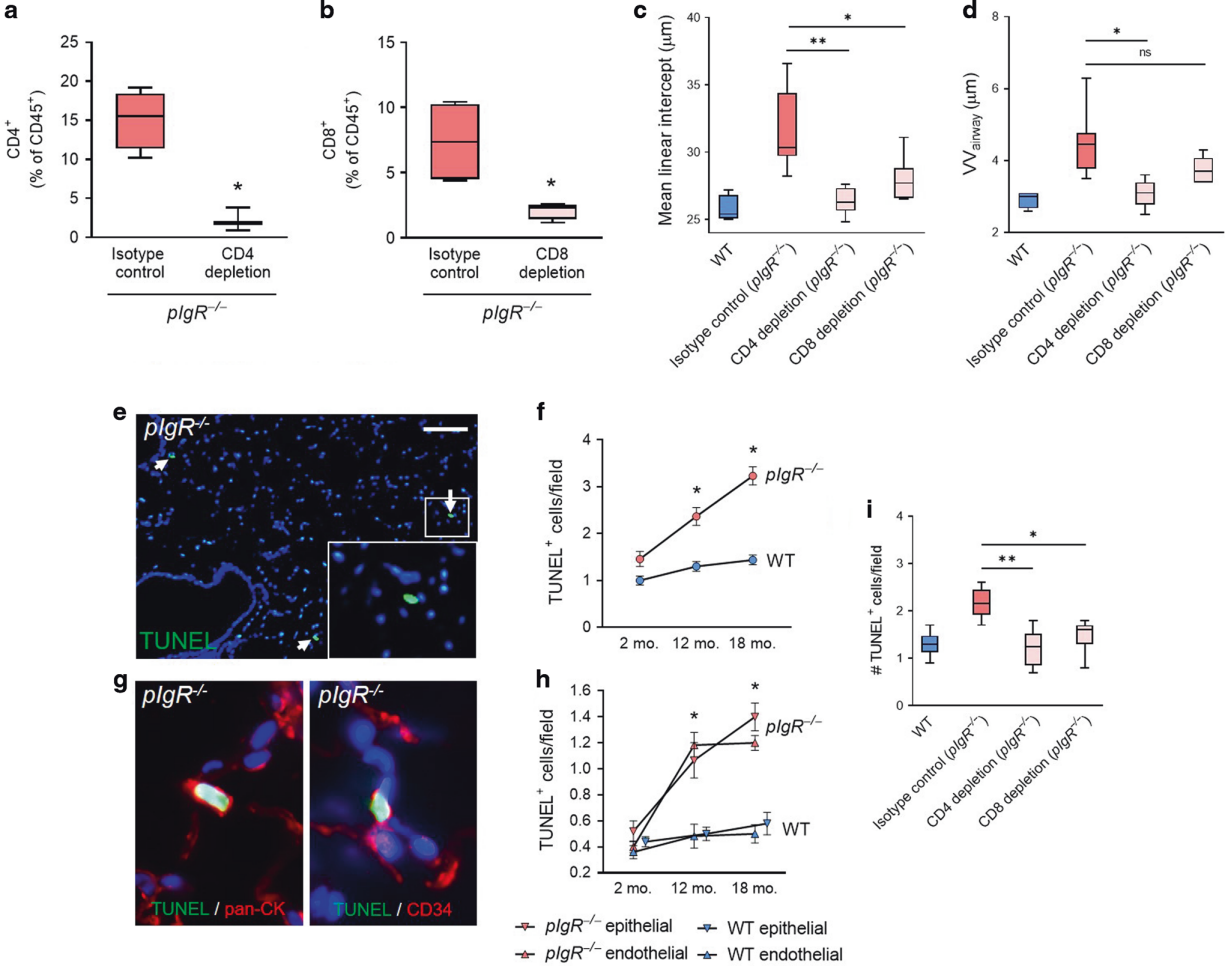
CD4^+^ and CD8^+^ T lymphocyte depletion blocks COPD-like pathology in *pIgR*^−/−^ mice and is associated with reduced apoptosis of lung epithelial and endothelial cells. **a**, **b** Percentage of CD4^+^ or CD8^+^ lymphocytes among CD45^+^ cells in spleens of mice treated with weekly intraperitoneal injection of anti-CD4 or anti-CD8 antibodies from 4 to 8 months of age. **c** Measurement of mean linear intercept (MLI), a morphometric measurement of emphysema, or **d** measurement of VV_airway_, a morphometric measurement of small airway wall thickness, in *pIgR*^*−/−*^ mice treated with anti-CD4 or anti-CD8 antibodies or rat IgG2b isotype control antibodies from 4 to 8 months of age. **e** TUNEL^+^ cells in the lung parenchyma of a *pIgR*^*−/−*^ mouse (white arrows denote TUNEL^+^ cells). Scale bar = 100 μm. **f** Quantification of TUNEL^+^ cells in *pIgR*^*−/−*^ and WT mice at the indicated ages. **g** Representative image of colocalization between TUNEL^+^ cells and pan-cytokeratin (marking epithelial cells) or CD34 (marking endothelial cells) in an 18-month-old *pIgR*^*−/−*^ mouse. **h** Quantification of TUNEL/pan-cytokeratin and TUNEL/CD34 dual-expressing cells in *pIgR*^*−/−*^ and WT mice at the indicated ages. **i** Quantification of TUNEL^+^ cells/field in 8-month-old *pIgR*^*−/−*^ mice treated with anti-CD8 or anti-CD4 antibodies or isotype control antibodies between 4 and 8 months of age. **a**, **b** *p < 0.05 compared to untreated mice (*t*-test); *n* = 3–4 mice*/*group. **c**, **d** *p < 0.05 com*p*ared to isotype-control treated mice, **p < 0.05 compared to isotype-control treated mice (1-way ANOVA with Bonferroni post hoc test); *n* = 4–8 mice/group. **f**–**h** *p < 0.001 compared to age-matched WT mice (2-way ANOVA with Bonferroni post hoc test); *n* = 5–6 mice/group. **i** *p < 0.01 compared to isotype control, *p < 0.0001 compared to isotype control; (1-way ANOVA with Bonferroni post hoc test); *n* = 7–8 mice/group. Box-and-whisker plots represent median, interquartile range, and range.

To investigate whether T lymphocytes contribute to COPD-like remodeling in *pIgR*^*−/−*^ mice, we treated these mice with weekly intraperitoneal injection of anti-CD8 antibodies (clone 2.43) or anti-CD4 antibodies (clone GK1.5) for 4 months. These treatments dramatically reduced splenic CD4^+^ and CD8^+^ T cells compared to isotype control rat IgG2b antibodies (clone LTF-2), confirming the effectiveness of this approach (Fig. 3a and b). In both CD4^+^ and CD8^+^ T lymphocyte-depleted animals, the effects of lymphocyte depletion were restricted to the target cell types (Supplementary Fig. 2). By histological analysis, *pIgR*^*−/−*^ mice treated with CD4-depleting antibodies had reduced emphysema and small airway wall thickening (Fig. 3c and d) compared to *pIgR*^*−/−*^ mice treated with isotype control. Similarly, *pIgR*^*−/−*^ mice treated with CD8-depleting antibodies had reduced emphysema and a trend toward reduced small airway wall thickness (Fig. 3c and d).

Since T lymphocytes are thought to stimulate apoptosis in the lung parenchyma in COPD patients,^19^ we investigated whether these cells contribute to the COPD phenotype in *pIgR*^*−/−*^ mice via this mechanism. Using terminal deoxynucleotidyl transferase (dUTP) nick end labeling (TUNEL) staining on lung sections from 2, 12, and 18-month-old WT and *pIgR*^*−/−*^ mice, we found that *pIgR*^*−/−*^ mice had increased numbers of TUNEL^+^ cells in the lung parenchyma at 12 and 18 months of age (Fig. 3e and f). Immunostaining for pan-cytokeratin (a marker of epithelial cells) and CD34 (a marker of endothelial cells) showed that both cell types were represented among TUNEL^+^ cells and that the proportion of TUNEL^+^ epithelial and endothelial cells was similar over time (Fig. 3g and h). To determine whether T lymphocytes contribute to increased apoptosis in *pIgR*^*−/−*^ mice, we measured numbers of TUNEL^+^ cells in lung sections from *pIgR*^*−/−*^ mice treated with anti-CD4 or anti-CD8 antibodies. We found that treatment with either CD4 and CD8-depleting antibodies reduced TUNEL^+^ cells to numbers similar to WT mice (Fig. 3i), indicating that increased apoptosis of structural cells in *pIgR*^*−/−*^ mice is T lymphocyte-dependent.

### Activated moDCs orchestrate T lymphocyte accumulation in the lungs of *pIgR*^*−/−*^ mice

**Fig. 4:**
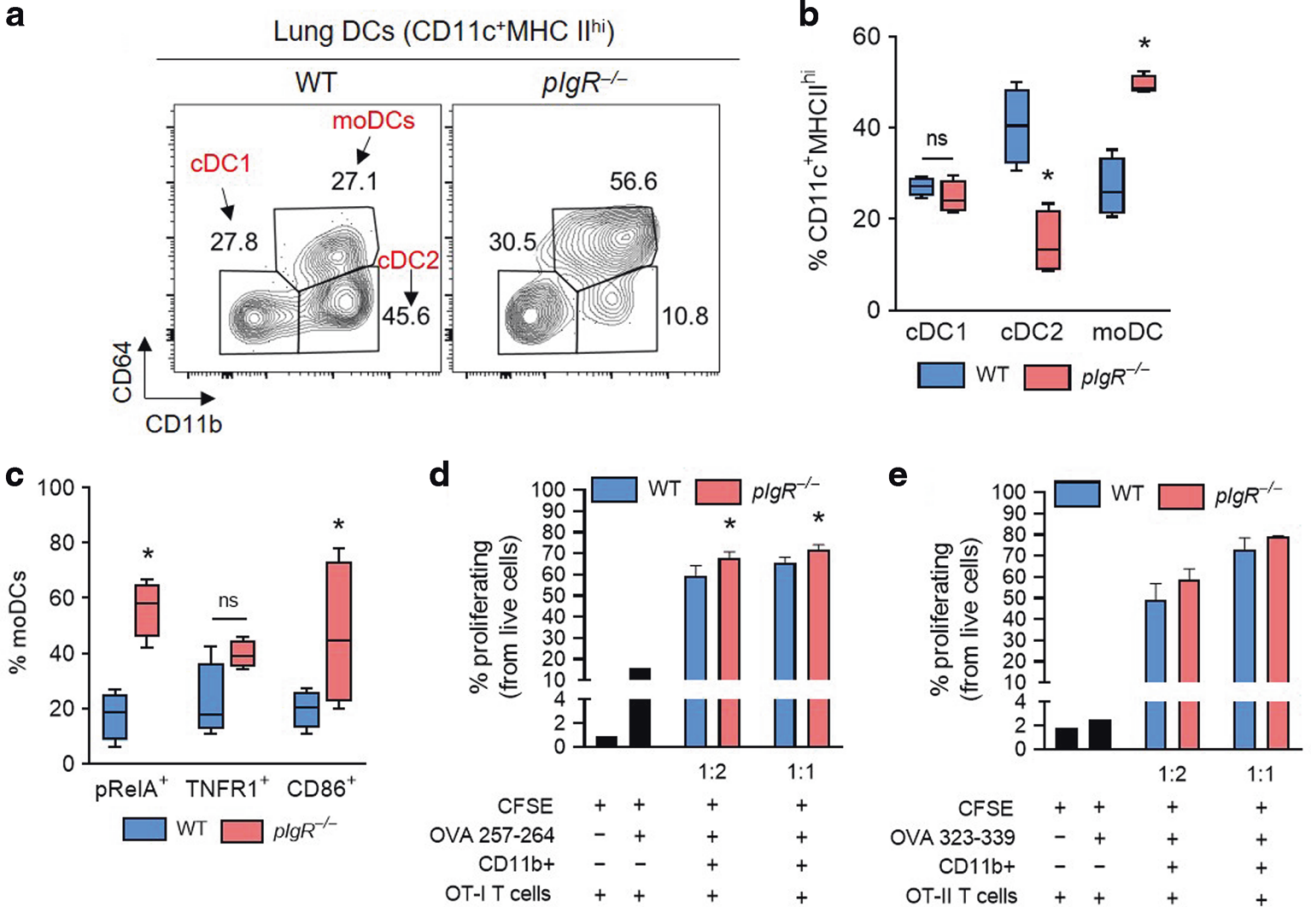
Activated moDCs accumulate in the lungs of *pIgR*^−/−^ mice. **a** Representative flow cytometry plot showing the distribution of cDC1, cDC2, and moDCs in the lungs of an 18-month-old WT and *pIgR*^*−/−*^ mouse (from CD11c^+^MHC II^hi^ cells). **b** Percentage of cDC1, cDC2, and moDCs among CD11c^+^MHCII^hi^ cells in 18-month-old WT and *pIgR*^*−/−*^ mice shown in (**a**). **c** Percentage of moDCs expressing the activation markers pRelA, TNFR1, and CD86 in 18-month-old WT and *pIgR*^*−/−*^ mice. **d**, **e** Percentage proliferating OT-I (**d**) or OT-II (**e**) splenic T lymphocytes co-cultured with lung CD11b^+^ cells from 18-month-old WT or *pIgR*^*−/−*^ mice at the ratios indicted (CD11b^+^ cells to T cells) in the presence of OVA peptide. **b**, **c** *p < 0.01 compared to 18-month-old WT mice (*t*-test); *n* = 4 mice/group. **d** *p < 0.05 for trend (1-way ANOVA); *n* = 3 biological replicates (CD11b^+^ cell donors)/group and 3 technical replicates per biological replicate. OT-I or OT-II cells came from spleens of a single donor for each experiment. Box-and-whisker plots represent median, interquartile range, and range.

DCs sample foreign antigens at mucosal surfaces and then migrate to draining lymph nodes where they promote the proliferation of naive and effector T cells.^25^ To determine if loss of SIgA impacts DC subsets in *pIgR*^*−/−*^ mice, we measured type 1 and 2 conventional and monocyte-derived DCs (cDC1, cDC2, and moDC) in 18-month-old WT and *pIgR*^*−/−*^ mice using an established flow cytometry protocol^26,27^ (Fig. 4a and Supplementary Fig. 3). Compared to aged WT mice, aged *pIgR*^*−/−*^ mice had reduced cDC2s and increased moDCs among the population of CD11c^+^MHCII^hi^ cells (Fig. 4b). Since moDCs have been shown to increase expression of pRelA, TNFR1, and CD86 in response to stimulation,^26^ we evaluated these markers and found an increased percentage of pRelA^+^ cells and a trend towards increased TNFR1^+^ and CD86^+^ moDCs in aged *pIgR*^*−/−*^ mice (Fig. 4c and Supplementary Fig. 4). To determine whether the DCs identified in these studies were functionally competent, we performed mixed lymphocyte reactions (MLR) using CD11b^+^ DCs isolated from the lungs of aged WT or *pIgR*^*−/−*^ mice and splenic-derived T cells from OT-I or OT-II mice which proliferate in response to ovalbumin peptides presented on MHC class I or II complexes, respectively.^28,29^ We found that CD11b^+^ DCs from aged *pIgR*^*−/−*^ and WT mice stimulated robust OT-I and OT-II T cell proliferation ex vivo in the presence of OVA peptides (Fig. 4d, e and Supplementary Fig. 5). Compared to WT mice, CD11b^+^ DCs from *pIgR*^*−/−*^ mice stimulated a slight increase in OT-I but not OT-II proliferation, suggesting that DCs from *pIgR*^*−/−*^ mice are competent to induce T cell proliferation (Fig. 4d and e).

**Fig. 5:**
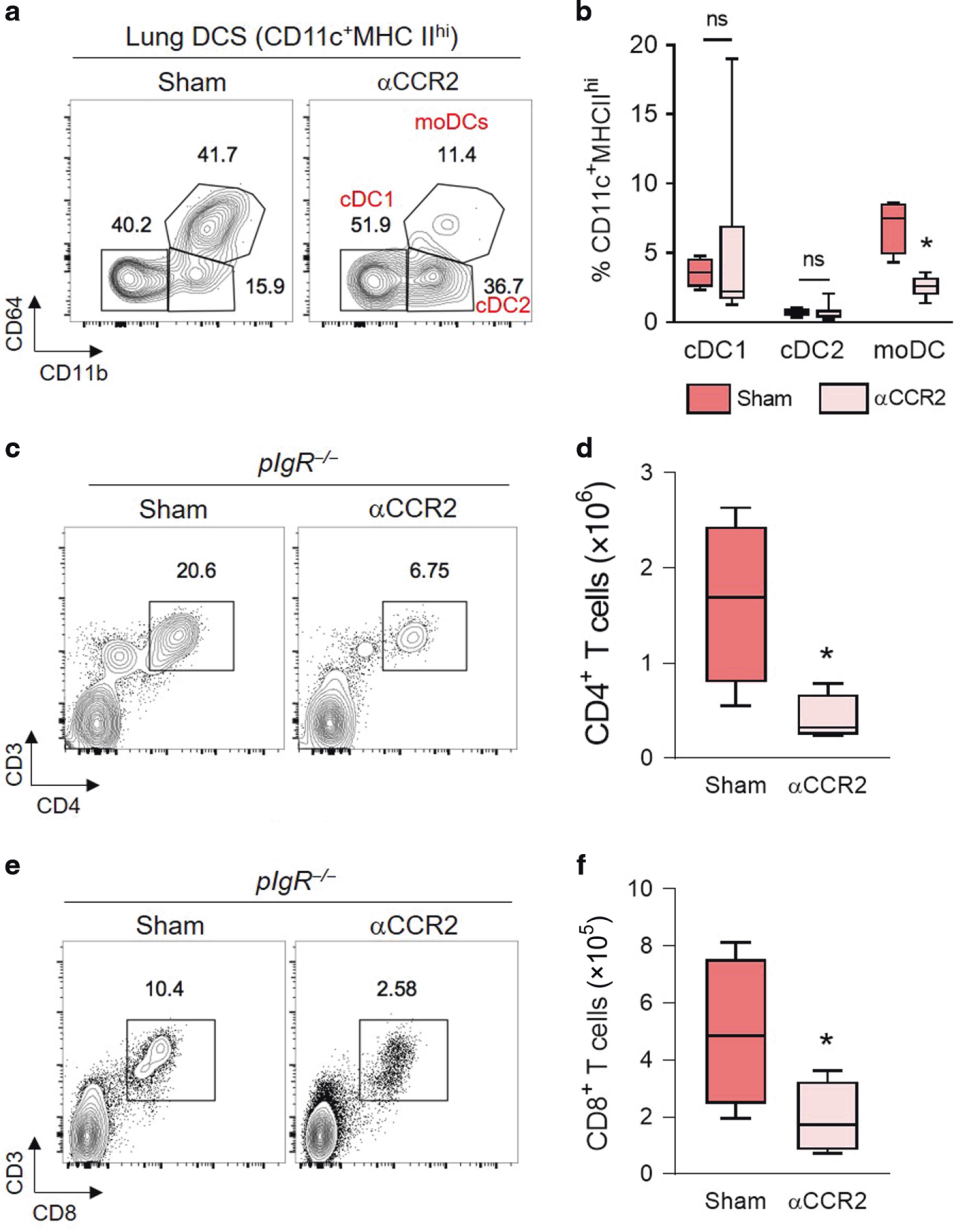
CCR2 blockade reduces moDC and T cell recruitment to the lungs of *pIgR*^−/−^ mice. **a** Flow cytometry plots demonstrating the effectiveness of the CCR2 antagonist RS-504393 in reducing lung moDCs in aged (20–22 months) *pIgR*^*−/−*^ mice. **b** Quantification of DC subsets (as % of CD11c^+^MHCII^hi^ cells) in the lungs of aged *pIgR*^*−/−*^ mice with and without CCR2 antagonist. **c**, **e** Representative flow cytometry plots showing CD4^+^ and CD8^+^ T lymphocytes in aged (20–22 months) *pIgR*^*−/−*^ mice with and without CCR2 antagonist treatment. **d**, **f** Quantification of CD4^+^ and CD8^+^ T lymphocytes shown in (**c**, **e**). **b**, **d** *p < 0.01 compared to sham-treated *pIgR*^*−/−*^ mice (*t*-test); *n* = 4–6 mice*/*group. **f** *p < 0.05 com*p*ared to sham-treated *pIgR*^*−/−*^ mice (*t*-test); *n* = 4–6 mice/group. Box*-*and-whisker plots represent median, interquartile range, and range.

CCR2 is the receptor for monocyte chemoattractant protein-1 (CCL2) and contributes to monocyte egress from the bone marrow to inflamed tissues. To determine whether moDCs contribute to increased numbers of T lymphocytes in SIgA-deficient mice, we treated aged *pIgR*^*−/−*^ mice with an orally available antagonist to CCR2 (RS-504393) that has been shown to block CCR2-dependent monocyte recruitment in vivo.^30^ After 2 weeks, we found that RS-504393 treatment resulted in a significant reduction in lung moDCs without affecting the number of cDC1 or cDC2 cells (Fig. 5a and b). Further, we found that RS-504393 treatment led to a marked reduction in lung CD4^+^ and CD8^+^ T lymphocytes (Fig. 5, c to f), supporting the idea that moDCs drive T cell recruitment in this model.

### Bacteria stimulate moDC-driven T lymphocyte recruitment in *pIgR*^*−/−*^ mice

**Fig. 6:**
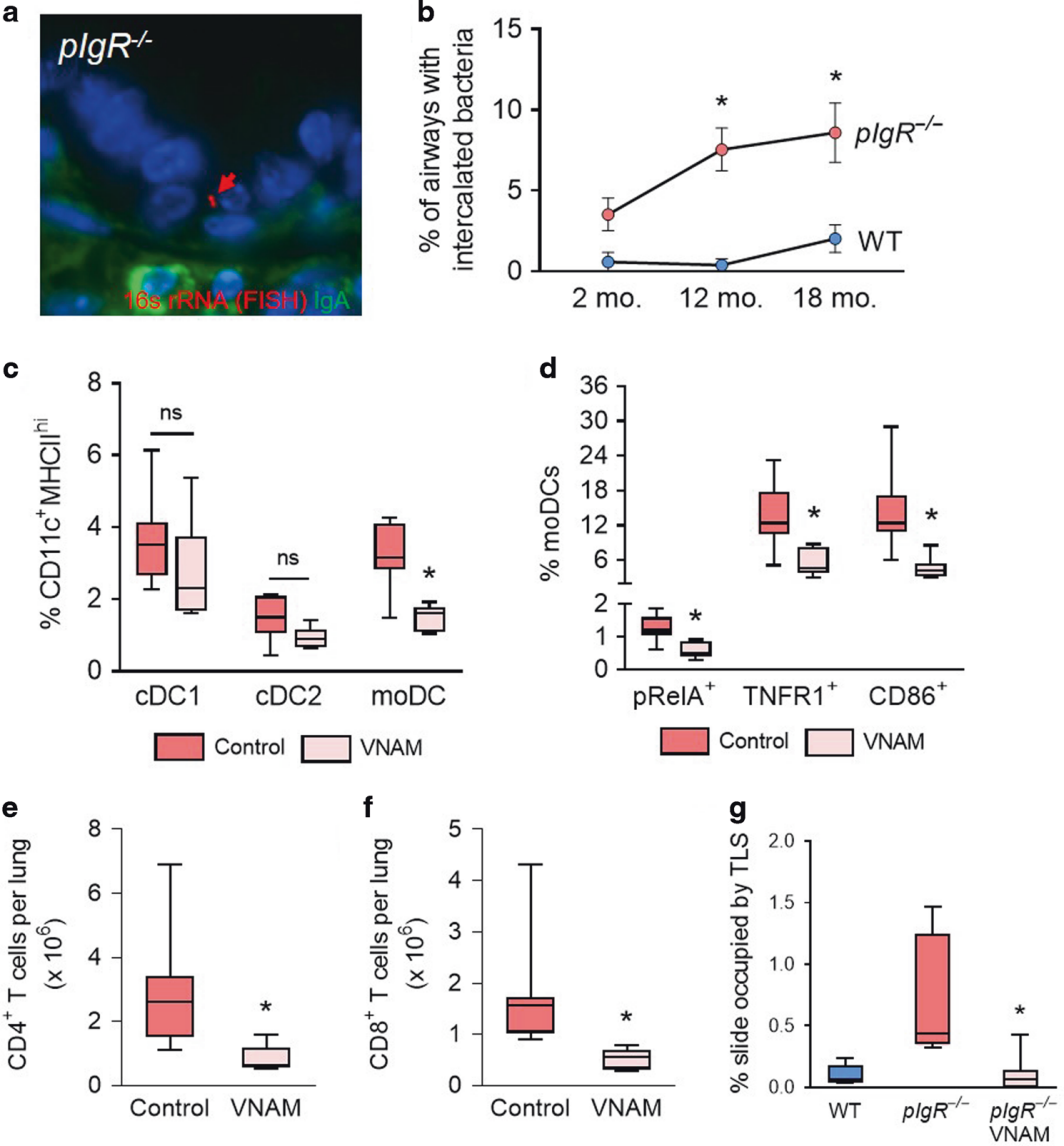
Antimicrobial therapy reduces activated moDC and T lymphocyte numbers in *pIgR*^−/−^ mice. **a** Representative in situ hybridization (FISH) of bacterium invading the epithelial barrier in a small airway from an 8-month-old *pIgR*^*−/−*^ mouse. The bacterium (red arrow) is labeled by a FISH probe targeting prokaryotic 16s rRNA. IgA staining (green) shows absence of SIgA on the airway surface. **b** Percentage of airways with bacteria intercalated into the mucosa in WT and *pIgR*^*−/−*^ mice at indicated ages. **c** Quantification of moDCs, cDC1, and cDC2 cells (as % CD11c^+^MHCII^hi^ cells) in aged *pIgR*^*−/−*^ mice with and without antibiotics (vancomycin, neomycin, ampicillin, metronidazole, VNAM) treatment. **d** Percentage of moDCs (CD11c^+^MHCII^hi^CD11b^+^CD64^+^) expressing the activation markers pRelA, TNFR1, and CD86 in the lungs of aged *pIgR*^*−/−*^ mice with and without antibiotic treatment. **e**, **f** Quantification of CD4^+^ and CD8^+^ T lymphocytes in the lungs of aged *pIgR*^*−/−*^ mice with and without antibiotic treatment. **g** Percentage of slide occupied by TLS in 18-month-old untreated WT and *pIgR*^*−/−*^ mice and 18-month-old *pIgR*^*−/−*^ mice treated with antibiotics. **b** *p < 0.01 compared to age-matched WT mice (2-way ANOVA with Bonferroni post hoc test); *n* = 5–11 mice*/*group. **c** *p < 0.01 com*p*ared to untreated *pIgR*^*−/−*^ mice (2-way ANOVA with Bonferroni post hoc test); *n* = 6–8 mice/group. **d**–**e** *p < 0.05 compared to untreated *pIgR*^*−/−*^ mice (*t*-test); *n* = 6–8 mice/group. Box-and-whisker plots represent median, interquartile range, and range. **f** *p < 0.001 compared to untreated *pIgR*^*−/−*^ mice (Mann–Whitney test); *n* = 6–8 mice/group. **g** *p < 0.01 compared to untreated *pIgR*^*−/−*^ mice (Mann–Whitney test); *n* = 6–8 mice/group.

Since SIgA is critical for airway defense against microbial invasion, we speculated that *pIgR*^*−/−*^ mice might be predisposed to increased bacterial invasion in the airways. Therefore, we identified bacteria within the airway epithelium of aged *pIgR*^*−/−*^ mice using a fluorescent in situ hybridization (FISH) probe directed against bacterial 16s rRNA (Fig. 6a). We found that bacterial invasion within the epithelial barrier of small airways was sustained up to 18 months of age in *pIgR*^*−/−*^ mice (Fig. 6b). We next investigated whether endogenous bacteria mediate moDC and T lymphocyte recruitment in the lungs of *pIgR*^*−/−*^ mice by treating mice with an antibiotic cocktail (vancomycin, neomycin, ampicillin, and metronidazole; VNAM) that previously has been shown to reduce bacterial burden in the lungs.^15^ *pIgR*^*−/−*^ mice treated with the VNAM cocktail between 15 and 18 months of age had reduced lung moDCs but no significant change in lung cDC1 or cDC2 cells (Fig. 6c). In addition, moDCs from *pIgR*^*−/−*^ mice treated with antibiotics also showed decreased expression of the moDC activation markers pRelA, TNFR1, and CD86 (Fig. 6d), suggesting that endogenous bacteria are responsible for moDC activation as well as recruitment. Further, we found that antibiotic treatment suppressed the influx of CD4^+^ and CD8^+^ T lymphocytes in the lungs and essentially eliminated TLS formation (Fig. 6, e to g). Taken together, these data elucidate a pathway in which loss of SIgA renders the airway epithelium more susceptible to bacterial invasion, which in turn stimulates moDC recruitment and activates a localized adaptive immune response.

### COPD patients have increased lung effector memory T lymphocytes and moDCs

To further interrogate T lymphocyte and dendritic cell responses in the lungs of COPD patients, we performed mass cytometry on single cell suspensions generated from the lungs of 12 patients with severe COPD and 6 organ donors whose lungs were rejected for transplantation (Supplementary Table 2). Using visualization of t-distributed stochastic neighbor embedding (viSNE),^31^ we identified T lymphocyte subsets defined by expression of CD3, CD4, and CD8 among total CD45^+^ cells from all samples (Supplementary Fig. 6A). We found increased numbers of CD4^+^ and CD8^+^ T lymphocytes among CD45^+^ cells in the lungs of patients with COPD (Supplementary Figs. 6B and C). To further determine the phenotypes of cells differentially present in COPD lungs, we performed a cluster identification, characterization, and regression (Citrus) analysis on T lymphocytes within the CD3^+^ island identified on viSNE. Citrus allows for unsupervised clustering of phenotypically similar cells followed by regularized supervised learning algorithms that identify the abundance of cells in each cluster according to clinical status (i.e., non-diseased control or COPD).^32^ The performance characteristics of our Citrus model are described in Supplementary Fig. 7. For Citrus analysis, we included markers to help distinguish naive versus memory T cells (CD45RO) and central versus effector memory (CCR7). Out of 33 lymphocyte clusters identified by Citrus (Supplementary Figs. 8), 4 had increased abundance in COPD lungs (Supplementary Figs. 6D) and 7 had lower abundance. Further analysis of the 4 upregulated clusters indicated one cluster of CD4^+^CD45RO^+^CCR7^+^ cells (CD4^+^ central memory), two clusters of CD4^+^CD45RO^+^CCR7^-^ cells which were distinguishable from each other only by their pattern of CD127 expression (CD4^+^ effector memory), and one cluster of CD8^+^CD45RO^+^CCR7^-^ cells (CD8^+^ effector memory) (Supplementary Fig. 6E). These findings indicate that increased numbers of antigen-responsive effector T cells are present in lungs of COPD patients.

**Fig. 7:**
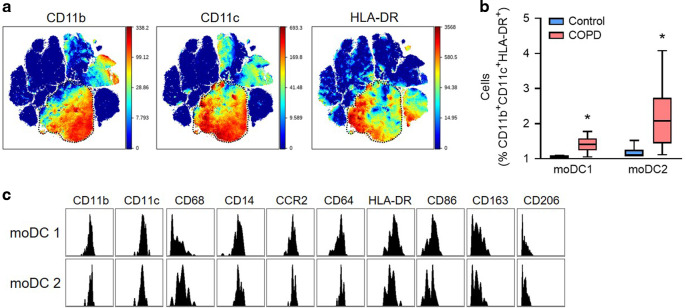
Increased moDCs in the lungs of COPD patients. Single-cell suspensions were prepared from the lungs of 12 COPD patients and 6 controls without chronic respiratory disease and analyzed by mass cytometry. **a** Expression of CD11b, CD11c, and HLA-DR in viSNE clusters generated from live, single, CD45^+^ cells from all patients (total of 400,000 cells). **b** Abundance (percentage among CD11b^+^CD11c^+^HLA-DR^+^ cells) of the 2 myeloid cell clusters enriched in COPD lungs relative to controls. **c** Histograms of myeloid cell markers in the 2 cell clusters (identified as moDCs) enriched in COPD lungs. *p < 0.01 compared to control lungs (Mann–Whitney test). Box-and-whisker plots represent median, interquartile range, and range.

We next used our mass cytometry panel to determine whether dendritic cell populations were phenotypically altered in the lungs of COPD patients. We performed a Citrus analysis on CD11b^+^CD11c^+^HLA-DR^+^ myeloid cells from the CD45^+^ viSNE island (Fig. 7a) and identified 29 separate cell clusters, only two of which were over-represented in COPD lungs (Fig. 7b, c and Supplementary Fig. 8). These two adjacent clusters expressed a combination of monocyte (CD14, CD64, CD68, CD86, and CCR2) and dendritic cell (HLA-DR, CD11b) markers, consistent with moDCs (Fig. 7c). The pattern of marker expression was very similar between the two upregulated clusters (putatively named moDC1 and moDC2), suggesting that they represent similar cellular phenotypes. Taken together, these data suggest that moDCs are uniquely over-represented among phagocytic cells in the lungs of COPD patients, consistent with findings in SIgA-deficient mice.

## Discussion

Our studies help to explain adaptive immune activation in COPD, which is common in patients with advanced disease. Our studies show that loss of the SIgA immunobarrier in individual small airways from patients with COPD is associated with CD4^+^ and CD8^+^ T lymphocyte infiltration and TLS formation around SIgA-deficient small airways. In SIgA-deficient (*pIgR*^*−/−*^) mice, we show that endogenous bacteria stimulate adaptive immune activation in the airways and that T lymphocytes directly contribute to emphysema and small airway wall thickening. Further, we show that moDCs are recruited to SIgA-deficient airways through a CCR2-dependent mechanism in response to airway bacteria, and these cells coordinate the pathologic adaptive immune response. Consistent with these findings, we identified increased moDCs in the lungs of patients with advanced COPD. Together, our studies suggest that loss of the SIgA immunobarrier is a fundamental defect driving adaptive immune activation in COPD.

Our findings that CD4^+^ and CD8^+^ effector memory cells are increased in the lungs of COPD patients are congruent with earlier work showing an increase in effector memory cells in the lungs of COPD patients compared to peripheral blood;^33^ however, we extended these findings to show increased numbers of these cells in the lungs of COPD patients relative to non-diseased control subjects. In our mouse model, we found that T lymphocytes were associated with increased apoptosis of parenchymal cells, which has been associated with emphysema in cross-sectional studies of COPD patients^19,34^ and other animal models.^35,36^ Activated CD8^+^ T lymphocytes can directly induce apoptosis through expression of perforin, granzymes, and Fas-ligand,^37^ while CD4^+^ T cells can contribute to apoptosis through the production of pro-apoptotic cytokines such as IFN-γ.^38^ However, it is also possible that apoptosis is indirect and mediated by other cell types in a T-cell dependent manner. Further studies will be required to clarify the mechanism of apoptotic cell death in this model.

Although moDCs have not previously been linked to human respiratory disease, our work indicates that moDCs are increased in COPD patients and link disruption of the SIgA immunobarrier to pathologic T cell responses. Since DCs sample and process exogenous antigens from the airways,^39^ these cells can serve as an important link between innate and adaptive immunity. While animal studies support a role for DCs in COPD pathology,^40–42^ studies examining DC subsets by immunohistochemistry or flow cytometry in COPD have yielded inconsistent results.^43^ We found that aged *pIgR*^*−/−*^ mice had increased moDC numbers and activation in the lungs as evidenced by increased pRelA, a marker of NF-κB activity.^44^ Reduction of the bacterial burden by antibiotic treatment blocked recruitment and activation of moDCs,^26^ indicating that airway bacteria are the primary stimulus for moDC differentiation in *pIgR*^*−/−*^ mice. Our data are consistent with other studies showing bacterial products stimulate moDC recruitment and maturation.^44,45^ While studies have shown the ability of monocytes to differentiate into dendritic cells in response to inflammation,^46–48^ the existence of moDCs in human tissues is not well established. In this regard, it was recently shown that a population of DCs that were transcriptionally similar to in vitro generated moDCs were increased in BAL fluid from healthy volunteers treated with nebulized bacterial lipopolysaccharide.^49^ Future studies are warranted to investigate phenotypes and functions of moDC subsets in human lungs.

Our work suggests that airway bacteria are the primary stimulus for moDC activation and T lymphocyte accumulation in SIgA-deficient airways. Airway bacteria have long been thought to contribute to COPD pathology^50^ and more recently have been implicated as a driver of TLS formation.^23,51^ While *pIgR*^*−/−*^ mice, like patients with COPD, do not appear to have bacterial overgrowth in the lungs,^14,52^ our data suggest that endogenous bacteria are more frequently localized within the epithelial layer, where they are capable of activating inflammatory signaling cascades. In the gut, it is clear that the binding affinity for SIgA differs among bacteria^53^ and directly modulates the composition of the microbiome.^54–56^ Conversely, the microbiome regulates both production and degradation of SIgA.^57^ Future studies will be required to understand how SIgA loss impacts the lung microbiome and whether individual bacteria differ in their ability to penetrate the mucosal immune barrier in SIgA-deficient small airways. Such investigations could lead to the development of probiotics aiming to restore the normal microbiome in SIgA-deficient airways.

We previously showed that neutrophils are important for the COPD-like pathology in lungs of *pIgR*^*−/−*^ mice but these cells appear prior to lymphocyte infiltration shown here.^14^ Although our work does not explain the temporal differences between innate and adaptive immune cell accumulation in this model, it is possible that neutrophils play a more prominent role in early disease, while lymphocytes are important in advanced disease. This idea is supported by human studies showing TLS are primarily observed in patients with advanced COPD.^20,58^ Potential interactions between T cells and neutrophils were also not fully addressed in our studies, although we observed a trend toward increased numbers of CD4^+^/IL-17A^+^ Th17 cells in *pIgR*^*−/−*^ mice. Since Th17 cells are known to contribute to lung destruction in animal models via effects on neutrophils^59,60^ and neutrophils are key mediators of COPD-like remodeling in this model,^15^ additional investigations regarding the role of Th17 cells in *pIgR*^*−/−*^ mice are warranted.

Our study has several limitations. Since we exclusively utilized COPD lung explants for human studies, our findings are only applicable to individuals with advanced disease. Further studies utilizing lung tissue from individuals with mild-moderate COPD will be required to understand whether moDCs accumulate in the lungs of COPD patients with early stage disease. Second, although CCR2 is known to recruit monocytes to inflamed tissues,^30^ it is also expressed by some CD4^+^ T lymphocytes.^61^ Therefore, we cannot exclude the possibility that CCR2 blockade had effects on CD4^+^ T lymphocytes aside from those mediated by moDCs. Third, we identified moDCs using a bulk analysis technique and did not document the location of these cells in situ. Because moDCs retain expression of multiple monocyte markers in addition to expressing markers of dendritic cells, multiplexed immunostaining or a similar technique will be required to document the location of these cells in vivo. Alternatively, future studies using scRNA-seq may be able to distinguish unique moDC markers that simplify their detection in situ. Finally, although B cells are increased in COPD patients^20,21^ and have been postulated to contribute to lung damage in COPD through the production of autoreactive antibodies,^62^ our study did not include an assessment of B cells in *pIgR*^*−/−*^ mice. Further studies will be required to clarify the role of B cells in this model.

In conclusion, we report that loss of the SIgA defense barrier results in bacterial invasion, moDC activation, and persistent activation of adaptive immunity in COPD patients. These studies provide a new paradigm for understanding adaptive immune activation in COPD and suggest new therapeutic targets for patients with this devastating disease.

## Methods

### Human studies

Human lung tissue was obtained from the explanted lungs of COPD patients undergoing lung transplantation at Vanderbilt University Medical Center and from organ donors whose lungs were rejected for various reasons. The clinical characteristics of these patients are described in Supplementary Tables 1 and 2. All patients provided written informed consent and the study protocol was approved by the institutional review board at Vanderbilt University Medical Center.

### Mice

All experiments were performed in accordance with protocols approved by the Institutional Animal Care and Use Committee of Vanderbilt University Medical Center. Male and female WT and *pIgR*^*−/−*^ mice (both C57Bl6 background) were maintained in standard microisolator cages on a 12-h light/dark cycle with food and water provided *ad libitum*. *pIgR*^*−/−*^ mice were obtained from the Mutant Mouse Resource Research Center at the University of Missouri, where they had previously been backcrossed onto a C57Bl6 background for a minimum of eight generations.^63^ For all experiments, WT and *pIgR*^*−/−*^ mice were housed separately. OT-I and OT-II mice (both C57Bl6 background) were obtained from the Jackson Laboratory and have been previously described.^28,29^

### Morphometry

All morphometric analyses were performed in accordance with the ATS/ERS Standards for Quantitative Assessment of Lung Structure.^64^ Measurements of small airway wall thickness (VV_airway_) and emphysema were performed as previously described.^12,14,20^ In mice, only cross-sectional distal airways, covered predominantly by secretory cells, were analyzed. Emphysematous changes of lung parenchyma were quantified using alveolar septal perimeter measurements and measurement of mean linear intercept on ten randomly chosen fields of alveolar tissue at ×200 original magnification. All morphometric measurements were made using Image-Pro Express software (Media Cybernetics, Silver Springs, MD).

### Lymphocyte depletion studies

Isoflurane-anesthetized *pIgR*^*−/−*^ mice were injected intraperitoneally with 200 μg of anti-CD8 (clone 2.43, BioXCell; catalog no. BE0061), anti-CD4 (clone GK1.5, BioXCell; catalog no. BE0003-1), or rat IgG2b isotype control (clone LTF-2, BioXCell; catalog no. BE0090) on week 1 followed by weekly injection of 100 μg of each antibody for 3 or 15 weeks as indicated. Antibodies were dissolved in sterile phosphate-buffered saline (PBS) in a total volume of 100 μl for each injection.

### Antibiotics and CCR2 antagonist administration

For antibiotics studies, vancomycin (0.5 mg/ml), neomycin (1 mg/ml), ampicillin (1 mg/ml), and metronidazole (1 mg/ml) were dissolved in autoclaved drinking water and provided to ~ 15-month-old *pIgR*^*−/*−^ mice *ad libitum* until euthanasia at 18 months of age. Water was changed twice weekly. Control animals received autoclaved water only. For CCR2 antagonist studies, ~18-month-old *pIgR*^*−/*−^ mice were treated by oral gavage with 2 μg RS504393 (Sigma, catalog no. SML0711) per gram mouse weight twice daily for 15 days. RS504393 was suspended in a 4% methylcellulose/water slurry. Sham-treated animals were gavaged with 4% methylcellulose/water slurry only.

### Generation of single cell suspensions

#### Murine lung

CO2-narcotized mice were euthanized via transection of the inferior vena cava. Lungs were perfused through the right ventricle with sterile PBS until white. Lungs were then removed, minced with scissors, and incubated for 45 minutes at 37 °C in RPMI 1640 medium containing collagenase XI (0.7 mg/mL; Sigma-Aldrich) and type IV bovine pancreatic DNase (30 μg/mL; Sigma-Aldrich). The tissue was further disrupted by gently pushing undigested pieces through a 70 μm nylon screen with the plunger from a plastic 5 ml syringe. Cells were then pelleted at 200 × *g*, incubated in 1 ml of ammonium-chloride-potassium (ACK) lysing buffer for 5 min at room temperature to remove red blood cells, washed with PBS containing 0.5% fetal bovine serum (FBS) and 2 mM EDTA, and filtered through a second 70 μm nylon screen.

#### Murine spleen

OT-I and OT-II mice were euthanized as described above. Spleens were removed, minced with scissors, and further disrupted by pushing small pieces through a 70 μm nylon screen with the plunger from a plastic 5 ml syringe. Cells were then pelleted at 200 × *g*, incubated in ACK lysing buffer as described above, washed with PBS containing 0.5% fetal bovine serum (FBS) and 2 mM EDTA, and filtered through a second 70 μm nylon screen.

#### Human lung

Tissue fragments were washing three times with sterile PBS to remove blood contamination. Fragments were minced with scissors in cold RPMI medium and then incubated for 45 min at 37 °C in RPMI 1640 medium containing collagenase XI (0.7 mg/mL; Sigma-Aldrich) and type IV bovine pancreatic DNase (30 μg/mL; Sigma-Aldrich). The tissue was further disrupted by gently pushing undigested pieces through a 70 μm nylon screen with the plunger from a plastic 5 ml syringe. Cells were then pelleted at 200 × *g*, incubated in 1 ml of ammonium-chloride-potassium (ACK) lysing buffer for 5 min at room temperature to remove red blood cells, washed with PBS containing 0.5% fetal bovine serum (FBS) and 2 mM EDTA, and filtered through a second 70 μm nylon screen. Cells were then pelleted, resuspended in FBS + 10% DMSO, and frozen at −20 °C for 24 h in an insulated cell freezer. Cells were then transferred to −80 °C where they remained until the time of mass cytometry experiments.

### Mixed lymphocyte reaction

CD11b^+^ cells from WT or *pIgR*^*−/−*^ mice were isolated via positive selection from lung single cell suspensions using immunomagnetic beads according to the manufacturer’s instructions (MACs Miltenyi; catalog no. 130-049-601). After isolation cells were maintained in RPMI 1640 medium supplemented with glutamine, 10% FBS, and 1% penicillin/streptomycin. T lymphocytes were isolated from single cell suspensions of OT-I or OT-II splenocytes using a negative-selection-based T cell enrichment column (R&D Systems; catalog no. MTCC-525). After isolation, T lymphocytes were labeled with the CellTrace CFSE Cell Proliferation Kit (Thermo-Fisher, catalog no. C34554). CD11b^+^ cells and OT-I or OT-II T lymphocytes were co-cultured in the presence of 1 μg/ml OVA peptide (OVA257-264 for OT-I, OVA323-339 for OT-II, both from AnaSpec) for 4 days at various ratios as indicated and proliferation determined by CFSE staining. Live cells were identified by DAPI exclusion.

### Flow cytometry

A listed of all antibodies utilized in this study can be found in Supplementary Table 3. The gating strategy for identifying DC subsets has previously been published^26,27^ and validated using DC-specific knock-out mice and DC-specific transcription factors.^26^ Briefly, single-cell lung suspensions were treated with Fc block (BD Pharmingen, purified anti-mouse CD16/CD32 Fc block, clone 2.4G2, catalog no. 553142) for 20 min on ice and then stained with fluorescent-dye-conjugated antibodies in PBS containing 2% FBS and 1 mM EDTA. Surface staining was performed at 4 °C fpr 20 min. Flow cytometry was performed on various instruments in the Flow Cytometry Shared Resource Center and at the University of Florida. All analyses were performed using FlowJo software.

### Histochemistry, immunohistochemistry, and TUNEL staining

5 μm serial sections were cut from each tissue specimen for hematoxylin and eosin and Masson trichrome staining. Additional 5 μm serial sections were used for immunostaining with the following antibodies: polyclonal rabbit anti-human IgA alpha chain (Agilent, catalog no. A026201-2, diluted 1:30); monoclonal (SP35) rabbit anti-human CD4 (Roche, catalog no. 790-4423, used undiluted); monoclonal (SP57) rabbit anti-human CD8 (Roche, catalog no. 790-4460, used undiluted); monoclonal (LE-CD19) mouse anti-human CD19 (GeneTex, catalog no. GTX42325, diluted 1:200); rat anti-mouse B220 (Invitrogen, catalog no. 14-0452-81, diluted 1:100); monoclonal (C-11) mouse anti-mammal pan-cytokeratin (Santa Cruz, catalog no. sc-8018); monoclonal (581) mouse anti-human CD34 (BioLegend, catalog no. 343502). Fluorescent in situ hybridization (FISH) was performed using a probe for the conserved bacterial 16S rRNA gene probe (Cy3GCTGCCTCCCGTAGGAGT-Cy3). TUNEL staining was performed using the In Situ Cell Death Detection Kit (Roche, catalog no. 11684795910) according to the instructions provided in the kit.

### Mass cytometry

Antibodies used for these studies can be found in Supplementary Table 4. Antibodies for mass cytometry or flow-cytometry were purchased pre-conjugated to a heavy metal (Fluidigm), biotin, or a fluorochrome (Biolegend). To identify dead cells, lung samples were previously incubated with 200 nM cisplatin (Sigma-Aldrich) for 5 min at room temperature (RT). Surface molecules were then stained with primary antibodies for 30 min at RT, followed by incubation with metal-conjugated secondary antibodies when needed. After surface staining, cells were fixed with 1.6% paraformaldehyde (PFA) in PBS for 20 min at RT, and then fixed and permeabilized with the Foxp3 fix/perm buffer set (Biolegend) for 45 min at RT. Cells were washed two times with perm wash, and incubated with anti-FOXP3, anti-CD68, anti-CD56, anti-CD4 and anti-CD8 (the surface markers used here were undetectable after tissue digestion, and an intracellular staining was used as an alternative method), for 30 min at RT. Cells were washed and resuspended in 1× iridium intercalator solution (Fluidigm) overnight. Next, samples were washed in PBS and then in distilled water and acquired using Helios mass cytometry at Vanderbilt University Mass Cytometry Center of Excellence. Acquisition was performed in the same day and all samples were normalized using five-element beads (Fluidigm).

### High-dimensional data analysis

To visualize different subpopulations of inflammatory cells in COPD and non-diseased control lungs, a two-dimensional viSNE-map was generated after gating live, single, and CD45^+^ leukocytes, based on event length, DNA (^191^Ir/^193^Ir), cisplatin (^195^Pt) and CD45 (^89^Y) staining. 400,000 cells were included in the final analyses and represent proportional down-sampling of 800,000 cells from all controls and COPD subjects. T lymphocytes (CD3^+^) or myeloid cells (CD11b^+^CD11c^+^ HLADR^+^) were gated from the map for further Citrus analysis using a Nearest Shrunken Centroid (PAMR) association model clustering cells by relative abundance (FDR = 1, cross validation folds=5, minimum cluster size=5%). Cell subpopulations differentially present in COPD patients were highlighted and the relative proportion was determined. The performance characteristics of the Citrus model, including cross-validation error rate and feature false discovery rates as a function of number of model features are given in Supplementary Fig. 5.

### Statistics

Mice were randomly assigned to the study groups and, where possible, researchers were blinded to the study groups until the time of statistical analysis. All animals were included in each analysis. Box and whisker plots show median, interquartile range, and range. Bar and line charts show mean ± standard error of the mean. For experiments involving repeated measurement of a single variable, one-way analysis of variance (ANOVA) with a Bonferroni post test was used. For experiments in which two variables were present, a two-way analysis of variance with a Bonferroni post test was used. Pair-wise comparisons were made using *t*-tests when the data was normally distributed and Mann–Whitney test when normality was not demonstrated. *p* < 0.05 was considered to be significant. Prism version 8 (GraphPad, San Diego, CA) was used for all statistical analyses.

## Supplementary information

Supplemental Fig and Table
