# Supplementary material for: Monocyte-derived dendritic cells link localized secretory IgA deficiency to adaptive immune activation in COPD
Source: Mucosal Immunol. 2020 Sep 23;14(2):431–42. doi: 10.1038/s41385-020-00344-9 (PMC7946625; doi:10.1038/s41385-020-00344-9)
Supplement: Supplementary file 1 — Supplemental Fig and Table [file 41385_2020_344_MOESM1_ESM.docx]

**Supplemental Figures**

**
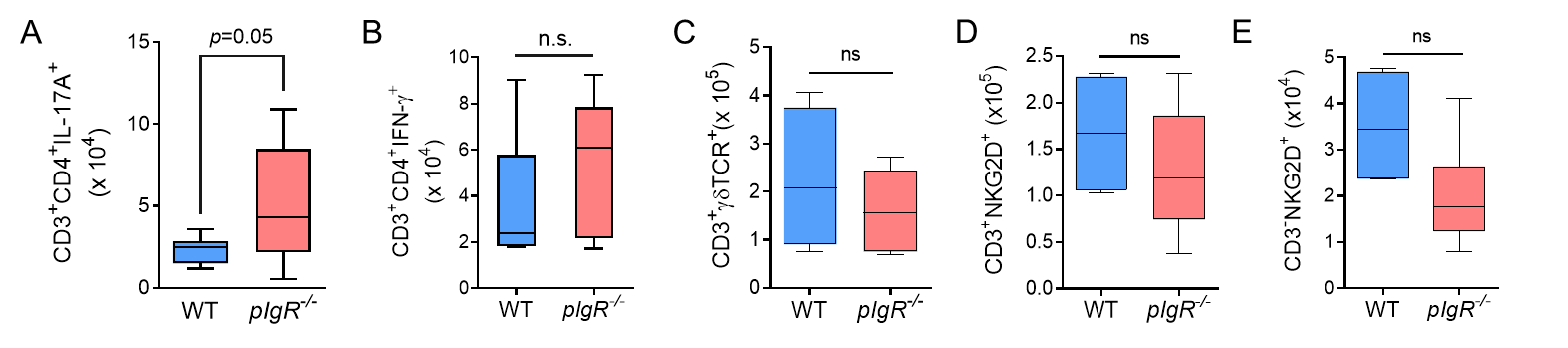
**

**Supplemental Figure 1. Quantification of IL-17A^+^ and IFN-γ^+^ CD4^+^ cells in the lungs of aged WT and *pIgR^-/-^* mice.** (**A**) Numbers of IL-17A^+^ CD4^+^ cells in lungs of 18-month-old WT and *pIgR^-/-^* mice as determined by flow cytometry. (**B**) Numbers of IFN-γ^+^ CD4^+^ cells in lungs of 12-month-old WT and *pIgR^-/-^* mice as determined by flow cytometry. (**C-E**) Numbers of γδ T cells (CD3^+^γδTCR^+^), NK−T cells (CD3^+^NKG2D^+^), and NK cells (CD3^-^NKG2D^+^) in lungs of 18-month-old WT and *pIgR^-/-^* mice as determined by flow cytometry. n.s. = not significant, *p*>0.05 by *t*-test.


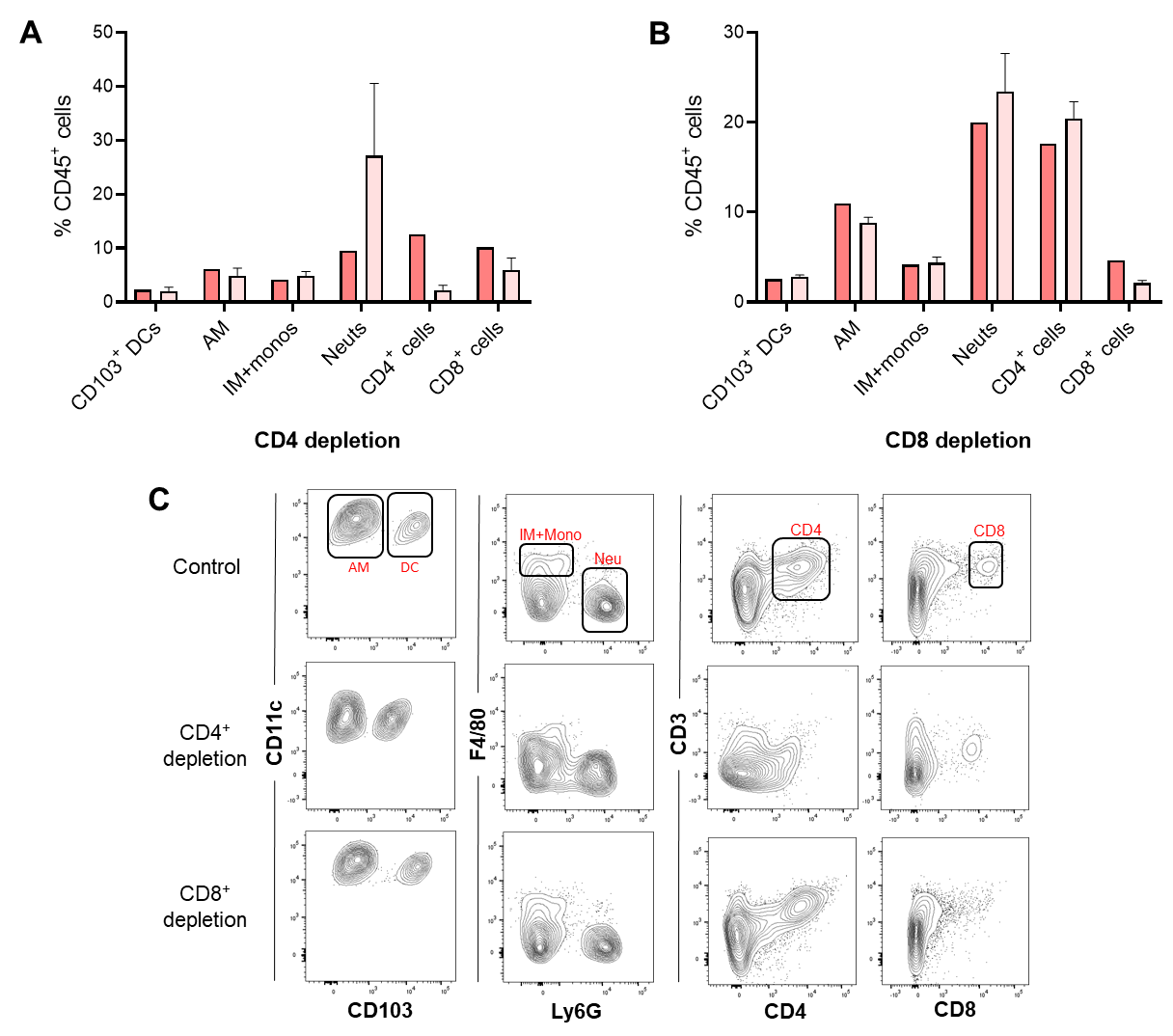


**Supplemental Figure 2. Effects of CD4 or CD8 depletion on other immune cell types in the lung.** Red denotes *pIgR^-/-^* control mice and pink denotes *pIgR^-/-^* mice treated with CD4 or CD8-depleting antibodies. (**A** to **B**) % immune/inflammatory cells among CD45^+^ cells in the lungs of *pIgR^-/-^* mice treated with CD4 or CD8-depleting antibodies for 1 month or no antibody. *n*=2-3 mice/group. (**C**) Gating strategy for **A** to **B**, including examples of flow cytometry plots in an untreated *pIgR^-/-^* mouse (top row), a *pIgR^-/-^* mouse treated with a CD8-depleting antibody for one month (middle row), and a *pIgR^-/-^* mouse treated with a CD4 lymphocyte-depleting antibody for one month (bottom row). CD45^+^ cells were the parent population for all plots. AM=alveolar macrophages, DC=dendritic cells, IM=interstitial macrophages, Monos=monocytes, Neu=neutrophils.


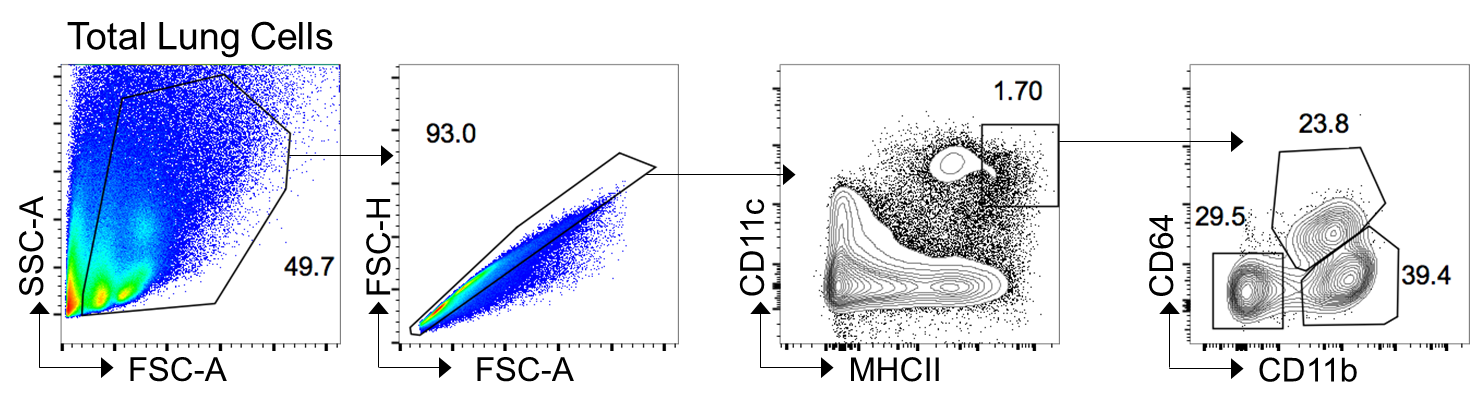


**Supplemental Figure 3. Back-gating for moDC/cDC1/cDC2 flow cytometry plots.** Gating strategy for identification of moDCs, cDC1, and cDC2 cells from total lung cells used in **Figure 4-6**.


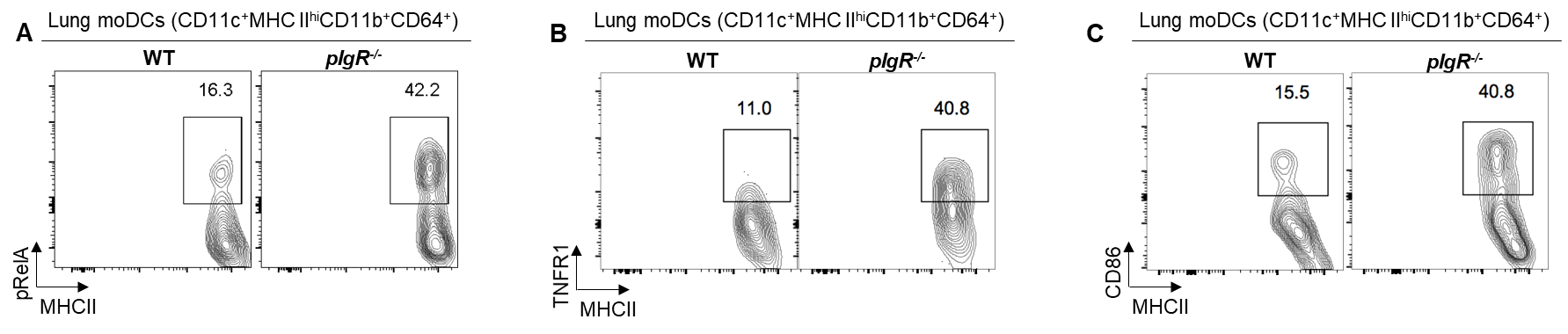

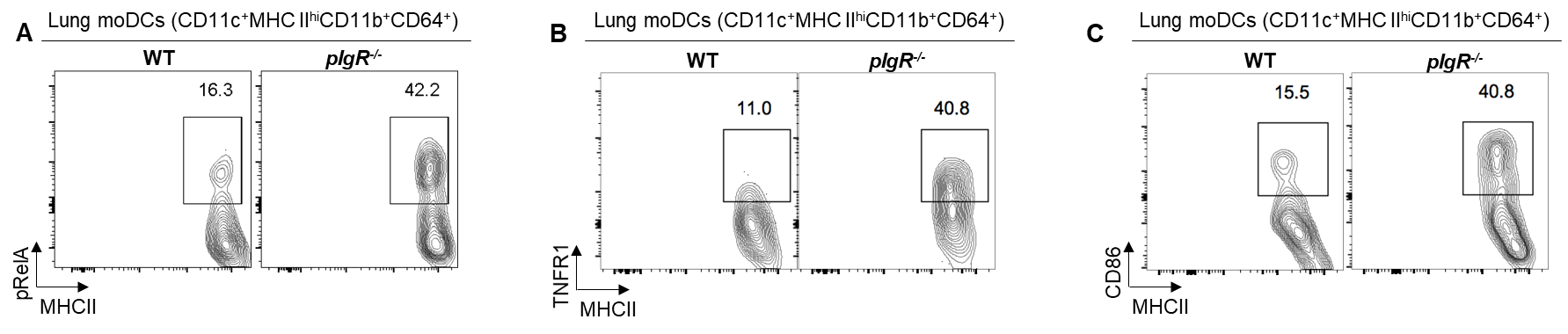

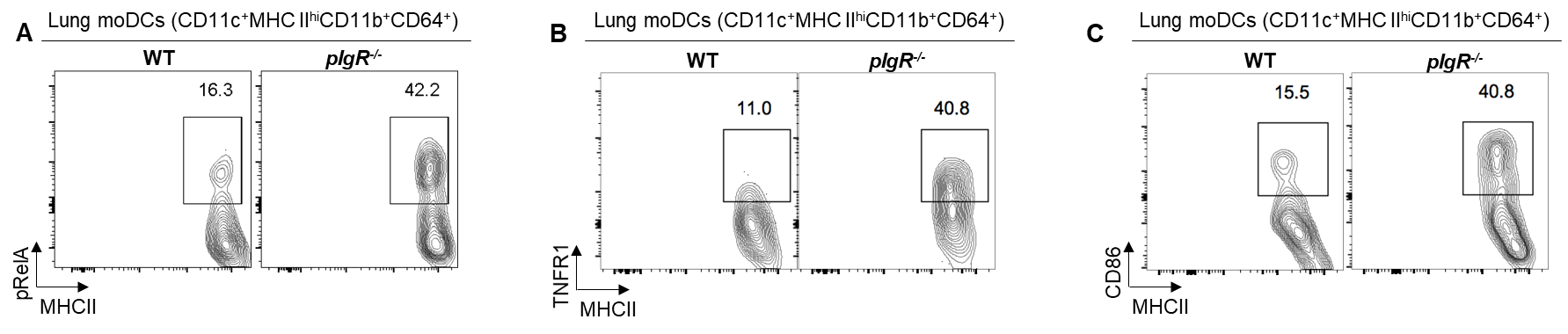


**Supplemental Figure 4. Representative flow cytometry plots for moDC activation markers.** These flow cytometry plots correspond to data shown in **Figure 4C.** (**A** to **C**) Representative flow cytometry plots for pRelA^+^, TNFR1^+^, and CD86^+^ cells in 18-month-old WT and *pIgR^-/-^* mice.

**
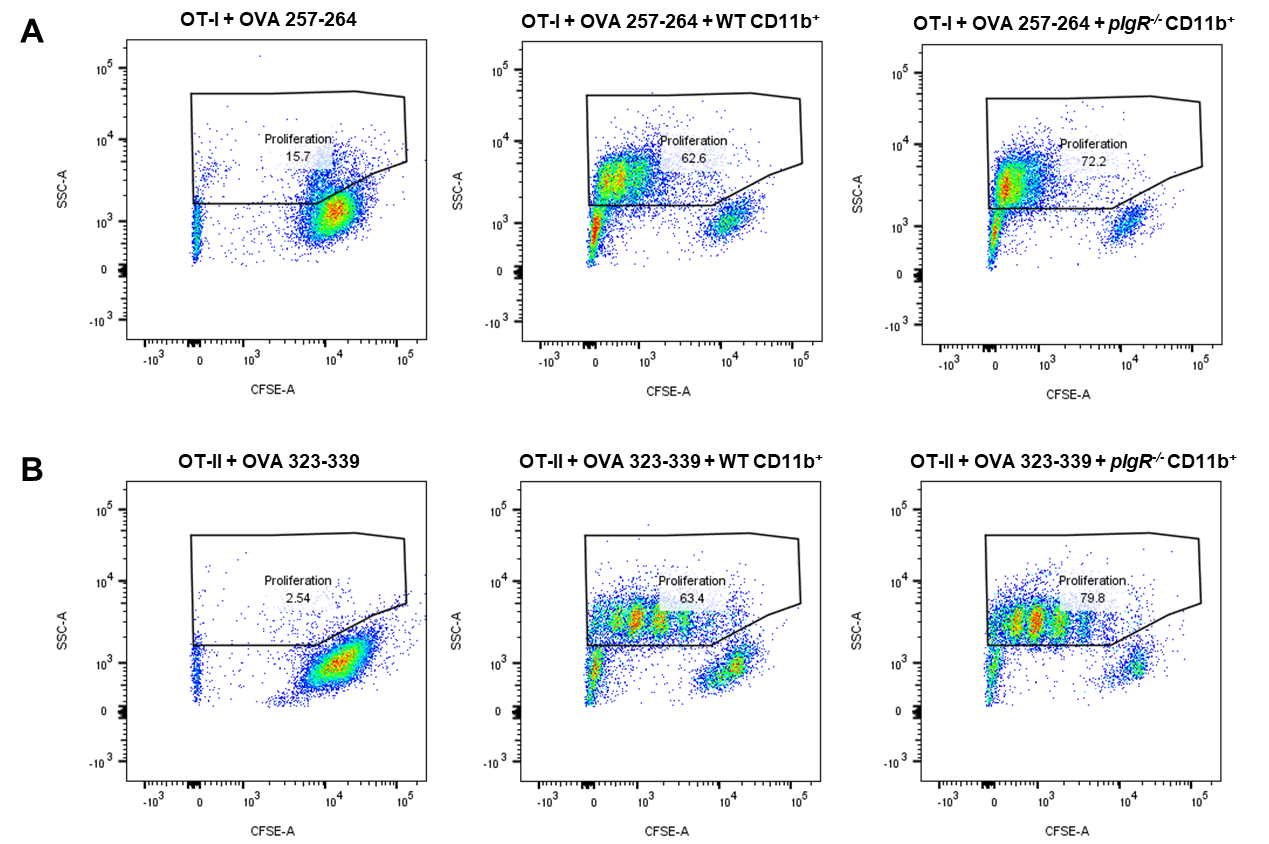
**

**Supplemental Figure 5. Representative flow cytometry plots for mixed lymphocyte reaction experiments.** These flow cytometry plots correspond to data shown in **Figure 4D and E**. (**A**) Representative flow cytometry plots showing OT-I proliferation by CFSE staining when cells were incubated in the presence of OVA 257-264 (left plot), OVA 257-264 and CD11b^+^ cells from 18-month-old WT mice (center panel), and OVA 257-264 and CD11b^+^ cells from 18-month-old *pIgR^-/-^* mice. (**B**) Representative flow cytometry plots showing OT-II proliferation by CFSE staining when cells were incubated in the presence of OVA323-339 (left plot), OVA323-339 and CD11b^+^ cells from 18-month-old WT mice (center panel), and OVA323-339 and CD11b^+^ cells from 18-month-old *pIgR^-/-^* mice.


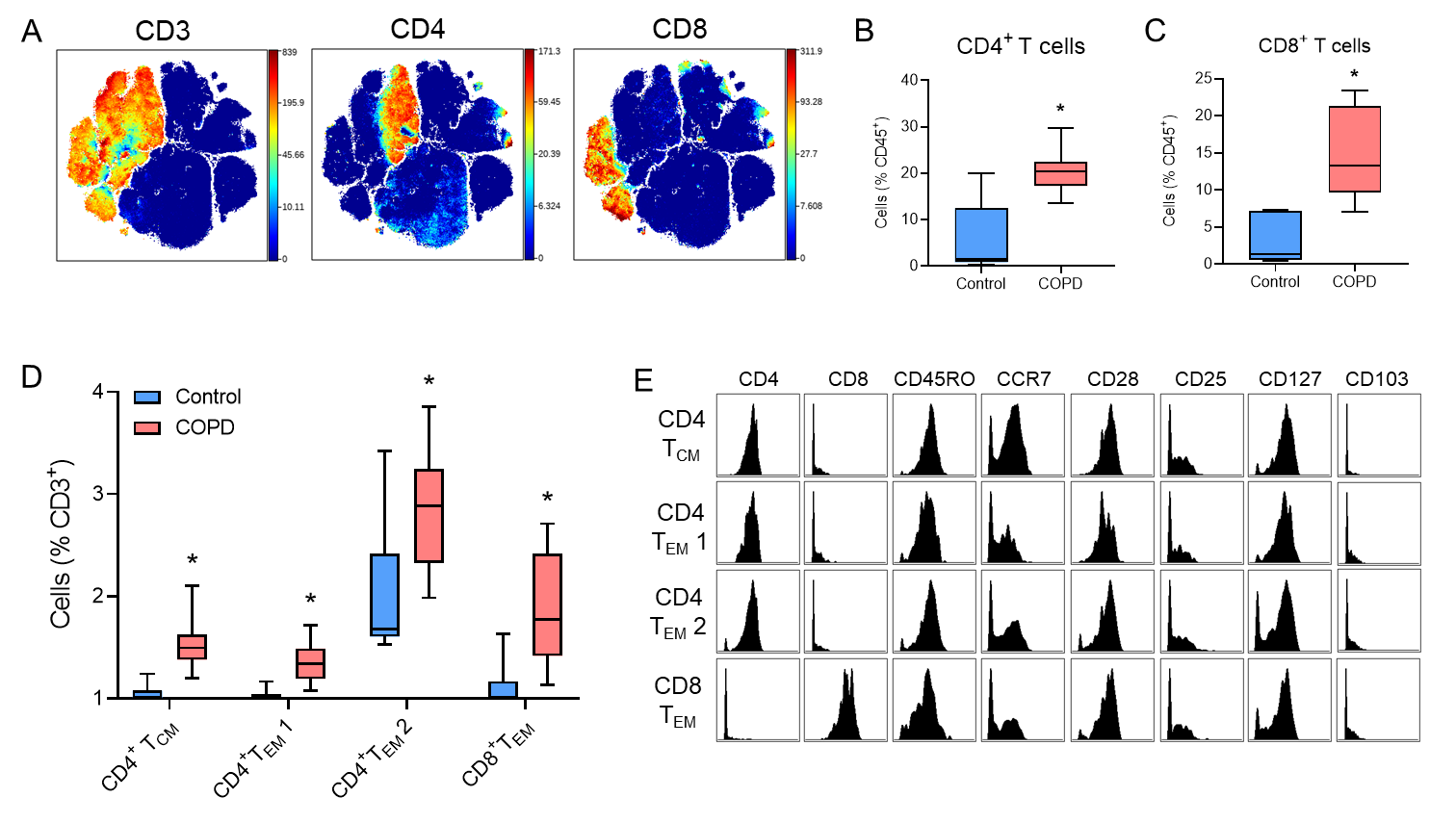


**Supplemental Figure 6. Increased effector memory CD4^+^ and CD8^+^ lymphocytes in the lungs of COPD patients.** Single-cell suspensions were prepared from the lungs of 12 COPD patients and 6 controls without chronic respiratory disease and analyzed by mass cytometry. (**A**) Expression of CD3, CD4, and CD8 in viSNE clusters generated from live, single, CD45^+^ cells from all patients (total of 400,000 cells. (**B** to **C**), Percentage of CD4^+^ and CD8^+^ cells among CD45^+^ cells in COPD and non-diseased control lungs. (**D**) Abundance (percentage of CD3^+^ cells) of the 4 lymphocyte clusters enriched in COPD lungs relative to controls. (**E**) Histograms of lymphocyte markers according to differentially abundant lymphocyte clusters as shown in **D**. (**B**), * = *p*<0.001 compared to control lungs (Mann-Whitney test); *n* = 12 COPD lungs and 6 control lungs. (**C**), * = *p*<0.001 compared to control lungs (*t*-test); *n* = 12 COPD lungs and 6 control lungs. (**D**) * = *p*<0.05 compared to control lungs (Mann-Whitney test).


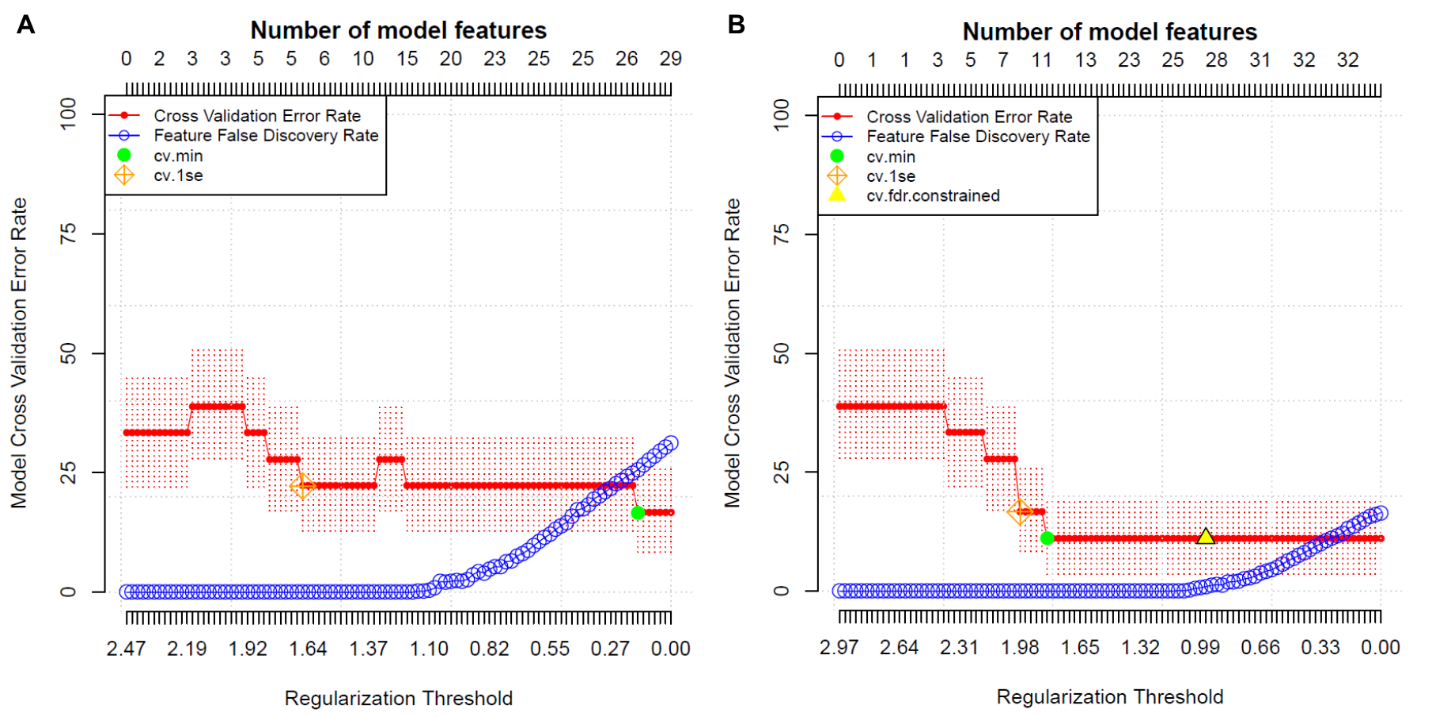


**Supplemental Figure 7. Performance characteristics of Citrus models.** These graphs correspond to the Citrus analyses described in **Figure 7-8**. The cross-validation error rate describes the ability of the Citrus model generated from all samples to describe behavior of a subset of samples. The cross-validation rate does not drop to zero because of unequal samples size between COPD and control samples. *cv.min* describes the model with the lowest cross-validation error rate and represents the minimum number of features needed to describe differential abundance in each cluster between COPD and control samples. *cv.1se* describes the model with the lowest cross-validation error rate within 1 standard error and also represents the minimum number of features needed to describe the differences between COPD and control samples. *cv.fdr.constrained* describes all of the different features below the false discovery rate (1%) and represents all the features required to describe differential abundance in each cluster between COPD and control samples.


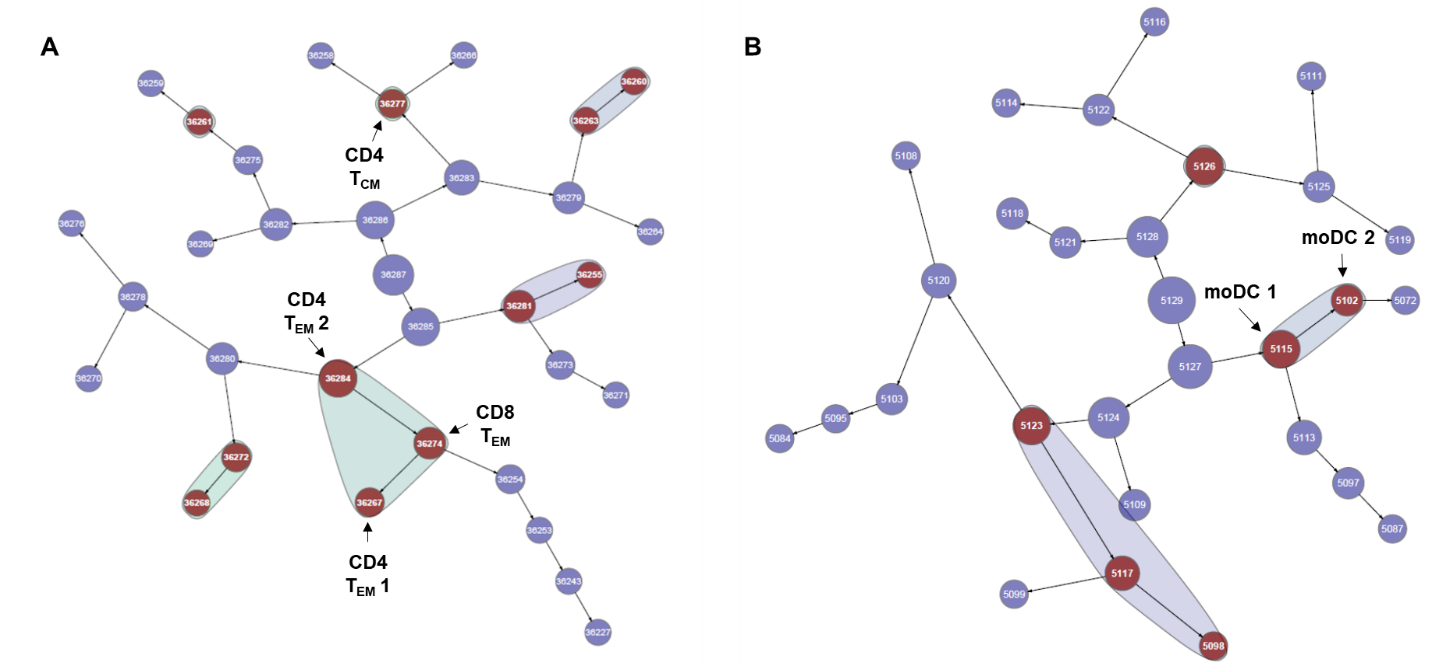


**Supplemental Figure 8. Citrus analyses for T lymphocyte and myeloid cell populations.** In this visualization, each cluster generated by Citrus is depicted by a single node, with the size of the node indicating the proportional abundance of the cluster and red nodes indicating differentially abundant clusters between COPD and control lungs. (**A**) Citrus analysis showing cell clusters within the lymphocyte (CD3^+^) viSNE islands. The labeled clusters were increased in COPD lungs. (**B**) Citrus analysis showing cell clusters within the myeloid cell (CD11b^+^CD11c^+^HLA-DR^+^) viSNE island. The labeled clusters were increased in COPD lungs.

**Supplemental Table 1.** Demographic and clinical characteristics of patients evaluated in **Figure 1**.

|  | **Lifelong non-smokers (*n*=8)** | **COPD**  **(*n*=12)** |  |
| --- | --- | --- | --- |
| **Age – yr.** |  |  |  |
| Mean | 61.9 | 56.7 |  |
| Range | 55-69 | 44-65 |  |
| **Sex – No. (%)** |  |  |  |
| Male | 5 (62.5) | 6 (50) |  |
| Female | 3 (37.5) | 6 (50) |  |
| **Race/ethnicity No. (%)** |  |  |  |
| White non-Hispanic | - | 11 (92.7) |  |
| Black | - | 1 (8.3) |  |
| **FEV1/FVC ratio** |  |  |  |
| Mean | 92.5 | 27.2 |  |
| Range | 61-117 | 21-42 |  |
| **FEV1, % predicted** |  |  |  |
| Mean | 101 | 20.9 |  |
| Range | 88-109 | 12-29 |  |
| **FVC, % predicted** |  |  |  |
| Mean | 79 | 63 |  |
| Range | 74-84 | 37-93 |  |
| **Tobacco history (pack-years)** |  |  |  |
| Mean | - | 38.8 |  |
| Range | - | 21-75 |  |
| **Alpha-1-antitrypsin deficiency** |  |  |  |
| No. (%) | - | 0 (0) |  |

**Supplemental Table 2.** Demographic and clinical characteristics of patients evaluated in **Figure 7 and 8**.

|  | **Control (*n*=6)** | **COPD (*n*=12)** |
| --- | --- | --- |
| **Age – yr.** |  |  |
| Mean | 35.5 | 57.8 |
| Range | 14-61 | 46-66 |
| **Sex – No. (%)** |  |  |
| Male | 1 (17) | 6 (50) |
| Female | 3 (50) | 6 (50) |
| Unknown | 2 (33) | 0 (0) |
| **Race/ethnicity** |  |  |
| White non-Hispanic | 3 (50) | 12 (100) |
| African American | 2 (33) | 0 (0) |
| Hispanic/Latino | 1 (17) | 0 (0) |
| **FEV1/FVC ratio** |  |  |
| Mean | - | 27.8 |
| Range | - | 21-34 |
| **FEV1, % predicted** |  |  |
| Mean | - | 16.5 |
| Range | - | 10-25 |
| **FVC, % predicted** |  |  |
| Mean | - | 46.1 |
| Range | - | 36-62 |
| **Tobacco history (pack-years)** |  |  |
| Mean | - | 45.7 |
| Range | 0-30 | 10-90 |
| **Current smoker** |  |  |
| No. (%) | 4 (66) | 0 (0) |
| **Alpha-1-antitrypsin deficiency** |  |  |
| No. (%) | - | 3 (25) |

**Supplemental Table 3.** List of flow cytometry antibodies.

| **Figure** | **Marker** | **Fluorophore** | **Clone** |
| --- | --- | --- | --- |
| Fig. 2 | CD45 | FITC | 30-F11 |
| Fig. 2 | CD3 | AF 700 | 17A2 |
| Fig. 2 | CD8 | BV 710 | 53-6.7 |
| Fig. 2 | CD4 | BV 510 | RM4-5 |
| Fig. 2 | CD19 | BV 570 | 6D5 |
| Fig. 2 | CD11c | APC | N418 |
| Fig. 2 | CD103 | PE | 2E7 |
| Fig. 2 | F4/80 | APC-Cy7 | BM8 |
| Fig. 2 | Ly6G | PE-Cy7 | IA8 |
| Fig. 5, 6 | CD3 | APC/Cy7 | 145-2C11 |
| Fig. 5, 6 | CD4 | PE/Cy7 | GK1.5 |
| Fig. 5, 6 | CD8 | APC | 53-6.7 |
| Fig. 4, 5, 6 | CD11c | APC/Cy7 | N418 |
| Fig. 4, 5, 6 | CD11b | PE/Cy7 | M1/70 |
| Fig. 4, 5, 6 | MHCII | Brilliant violet 421 | M5/114/15.2 |
| Fig. 4, 5, 6 | CD64 | PerCP/Cy5.5 | X54-5/7.1 |
| Fig. 4, 5, 6 | pRelA | APC | 93H1 |
| Fig. 4, 5, 6 | TNFR1 | PE | HM104 |
| Fig. 4, 5, 6 | CD86 | APC/Cy7 | GL-1 |
| Fig. S1 | Live/dead | Ghost UV 450 |  |
| Fig. S1 | CD45 | Alexa Fluor 700 | 30-F11 |
| Fig. S1 | CD3 | BV786 | 145-2C11 |
| Fig. S1 | CD4 | PE-Cy5 | 129.19 |
| Fig. S1 | IL-17A | PE-Cy7 | eBio17B7 |

**Supplemental Table 4.** List of mass cytometry antibodies.

| **Figure** | **Marker** | **Metal conjugate** |
| --- | --- | --- |
| Fig. 7 and 8 | CD45 | 89Y |
| Fig. 7 and 8 | CD19 | 142Nd |
| Fig. 7 and 8 | CD11b | 144Nd |
| Fig. 7 and 8 | CD4 | 145Nd |
| Fig. 7 and 8 | CD8a | 146Nd |
| Fig. 7 and 8 | CD11c | 147Sm |
| Fig. 7 and 8 | CD16 | 148Nd |
| Fig. 7 and 8 | CD127 | 149Sm |
| Fig. 7 and 8 | CD86 | 150Nd |
| Fig. 7 and 8 | HLA-DR | 151Eu |
| Fig. 7 and 8 | CD36 | 152Sm |
| Fig. 7 and 8 | CCR4 | 153Eu |
| Fig. 7 and 8 | CD163 | 154Sm |
| Fig. 7 and 8 | CD169 | 158Gd |
| Fig. 7 and 8 | FOXP3 | 159Tb |
| Fig. 7 and 8 | CD14 | 160Gd |
| Fig. 7 and 8 | CD103 | 161Dy |
| Fig. 7 and 8 | CD28APC +anti-APC | 162Dy |
| Fig. 7 and 8 | CD34 | 163Dy |
| Fig. 7 and 8 | CD45RO | 164Dy |
| Fig. 7 and 8 | CD64-PE + anti-PE | 165Ho |
| Fig. 7 and 8 | CD24 | 166Er |
| Fig. 7 and 8 | CCR7 | 167Er |
| Fig. 7 and 8 | CD206 | 168Er |
| Fig. 7 and 8 | CD25 | 169Tm |
| Fig. 7 and 8 | CD3 | 170Er |
| Fig. 7 and 8 | CD68 | 171Yb |
| Fig. 7 and 8 | CD38 | 172Yb |
| Fig. 7 and 8 | CCR2 FITC+ anti-FITC | 174Yb |
| Fig. 7 and 8 | CD56 | 176Yb |
| Fig. 7 and 8 | Nuc acid --Ir | 191/193 |
| Fig. 7 and 8 | Cisplatin | 198Pt |
